# IL20RA signaling enhances stemness and promotes the formation of an immunosuppressive microenvironment in breast cancer

**DOI:** 10.7150/thno.45280

**Published:** 2021-01-01

**Authors:** Wenjuan Gao, Huiping Wen, Luyu Liang, Xiaoli Dong, Renle Du, Wei Zhou, Xuehui Zhang, Chunze Zhang, Rong Xiang, Na Li

**Affiliations:** 1 School of Medicine, Nankai University, 94 Weijin Road, Tianjin, PR China.; 2 Tianjin's Clinical Research Center for Cancer, Tianjin, PR China.; 3 Key Laboratory of Breast Cancer Prevention and Therapy, Tianjin Medical University, Ministry of Education, Tianjin, PR China.; 4 Department of Blood Transfusion, Tianjin Medical University Cancer Institute and Hospital, Tianjin, PR China.; 5 Department of Colorectal Surgery, Tianjin Union Medical Centre, Tianjin, PR China.; 6 Tianjin Key Laboratory of Tumour Microenvironment and Neurovascular Regulation, Tianjin, PR China.

**Keywords:** IL20RA, SOX2, breast cancer, stemness, nanoparticles

## Abstract

**Rationale:** Tumor microenvironment interacts with tumor cells to regulate their stemness properties through various cytokines and cytokine receptors. Previous studies revealed the possible role of interleukin 20 receptor subunit alpha (IL20RA) signaling in the progression of several types of tumors. However, its regulatory effects on the stemness and the microenvironment of breast cancer need to be studied.

**Methods:** Immunohistochemical staining and western blot analysis were used to evaluate the association between IL20RA and SOX2 in breast tumors and noncancerous tissues. Enzyme-linked immunosorbent assay and TCGA dataset analysis were performed to determine the function of IL20RA signaling in breast cancer progression. Gain- and loss-of-function methods were performed to examine the effects of IL20RA on the stemness of breast cancer cells. The stemness features were analyzed by detecting the expression of core stemness genes, side population (SP), sphere formation ability, and aldehyde dehydrogenase (ALDH) activity. Flow cytometric analysis was applied to detect the changes of tumor-infiltration lymphocytes in tumor tissues in mice. Based on the relevant molecular mechanisms elucidated in this study, a novel IL20RA-targeted liposomal nanoparticle encapsulating the signal transducer and activator of transcription 3 (STAT3) inhibitor stattic (NP-Stattic-IL20RA) was synthesized. These NPs were combined with anti-programmed death ligand 1 (PD-L1) antibody and chemotherapy to inhibit the development of breast tumors in mice.

**Results:** IL20RA is highly expressed in human breast cancers and is positively associated with the SOX2 expression. IL20RA increases the SP and ALDH^br^ proportions of breast cancer cells, enhances the sphere formation ability, and promotes the expression of core stemness genes, such as *Sox2* and *Oct4*, as well as increases chemoresistance of breast cancer cells. IL20RA promotes the tumor-initiating ability and lung metastasis of breast cancer cells *in vivo*. In addition, IL20RA activates the Janus kinase 1 (JAK1)-STAT3-SOX2 signaling pathway, leading to increased expression of PD-L1 and reduced recruitment of anti-cancer lymphocytes, including CD8^+^ T cells and natural killer cells. Meanwhile, IL20RA signaling enhances the proportion of myeloid-derived suppressor cells. Combined with anti-PD-L1 antibody and NPs-Stattic-IL20RA, the chemotherapeutic efficacy was increased in breast cancer mouse models* in vivo*.

**Conclusion:** Collectively, our results reveal that the IL20RA pathway is a novel signaling pathway involved in promoting the stemness features of breast cancer along with the formation of a tumor-favorable immune microenvironment. Targeting the IL20RA^hi^ population with STAT3 signaling inhibition combined with anti-PD-L1 antibody can increase the therapeutic efficacy of chemotherapeutic agents for breast cancer. This study thus introduces a promising novel strategy for breast cancer therapy.

## Introduction

Recent studies have revealed the vital role of cancer stem cells (CSCs) in tumor initiation, recurrence, metastasis and resistance to conventional chemotherapy and radiotherapy 1, 2. CSCs have been identified in many types of cancer, including leukemia 3, colon cancer 4, and breast cancer 5. The discovery of CSCs inspires the development of novel CSCs-targeted therapeutic strategies.

The transcription factor SOX2 plays an important role in maintaining the stemness properties and apoptosis resistance of cancer cells 6-9. SOX2 is ectopically expressed in various cancers and is a prognostic marker and a promising therapeutic target in cancer 10. The tumor microenvironment interacts with tumor cells and regulates their stemness through many factors, including cytokines, growth factors. Several cytokine/cytokine receptor pathways are reported to regulate the stemness properties of breast cancer through regulating the expression of SOX2 11.

Interleukin 20 receptor subunit alpha (IL20RA) belongs to the type II cytokine receptor family. Upon binding to its ligands, such as interleukin (IL)-19, IL-20, and IL-24, IL20RA can form a functional heterodimeric receptor with IL20RB 12, 13. IL-26, another IL20RA ligand, requires IL20RA and interleukin 10 receptor subunit beta (IL10RB) for signaling 12, 14. *IL20RA* and *IL20RB* are mainly co-expressed in the skin and testis 13 . These ligands belong to the IL-10 family and the IL-20 subfamily of cytokines. IL-19, IL-20 and IL-24 are primarily expressed in monocytes, while memory T cells and natural killer (NK) cells are the primary cellular sources of IL‑26 15. These ligands and IL20RA are implicated in multiple inflammatory diseases, including rheumatoid arthritis, psoriasis, and Crohn's disease 13, 16-19.

Current studies provide evidences that IL20RA signaling regulates the development of cancer. Previously, IL-20 was reported to promote the progression of prostate cancer, oral cancer, and breast cancer 20-22. IL-19 and IL-20 are highly expressed in breast cancer and are associated with a poor clinical outcome 22, 23. IL-24 inhibits tumor cell growth by inducing apoptosis and/or cell cycle arrest in several types of cancer, including leukemia, breast cancer, and pancreatic cancer 24-26. IL-24 also inhibits the migration of lung and pancreatic cancer cells 27, 28. IL-26 promotes the growth of gastric cancer and is reported to be a risk factor for this cancer type 29, 30. It was reported that silencing of the *IL20RA* gene via promoter hypermethylation may promote the development of lung cancer 31. Upon binding to its ligands, IL20RA activates Janus kinase-signal transducer and activator of transcription (JAK-STAT) signaling 12. However, its role in the regulation of breast cancer stemness and progression remains to be further studied.

Programmed death ligand 1 (PD-L1) and programmed cell death 1 (PD-1) play important roles in mediating immunosuppression during the development of cancer 32, 33. Previous reports demonstrated high expression of PD-L1 in various human solid tumors, including lung cancer, melanoma, ovarian cancer, and colon cancer 33. However, with the exception of macrophages in the tonsil, liver, and lung tissues, its expression is almost undetectable in normal tissues 33. PD-L1 promotes the apoptosis of activated tumor antigen-specific T cells both *in vitro* and* in vivo*, thereby suppressing the anti-tumor immune response. PD-L1 is upregulated in multiple cancer types, and the PD-L1/PD-1 interaction between tumor cells and T cells leads to immunosuppression 33, 34. Immune checkpoint inhibitors, such as anti-PD-1 or anti-PD-L1 antibodies, can reactivate the activity and proliferation of T cell and stimulate immune cells to recognize and eradicate cancer cells 35. The response rate of PD-1/PD-L1-blocking therapeutics is much higher in patients whose tumors exhibit PD-L1 expression than in patients without tumor PD-L1 expression 36. Previous reports revealed high levels of PD-L1 in CSCs-like population of several tumors, including colorectal cancer and lung squamous cell carcinoma 37, 38. These findings suggest a potential mechanism for CSCs to evade immune surveillance.

In this study, the roles of IL20RA in breast cancer progression and stemness were investigated. We found that IL20RA promotes stemness features and increases the tumor-initiating ability of breast cancer cells via the JAK1-STAT3-SOX2 signaling pathway. Moreover, IL20RA promotes the chemoresistance of breast cancer cells and upregulates the expression of PD-L1 to compromise the activity of anti-cancer immune cells. Chemotherapy combined with PD-L1 blockade and IL20RA-targeted delivery of a STAT3 inhibitor is highly effective in the treatment of breast tumors. This is thus a promising novel combined therapeutic strategy for breast cancer.

## Material & Methods

### Immunohistochemistry staining

Immunohistochemistry (IHC) staining was performed on a human breast tissue array (Alenabio Company, Xi'an, Shanxi, China) using primary antibodies raised against either IL20RA or SOX2 (Abcam, Cambridge, UK). The clinical characteristics of patients were previously described 39.

IHC staining was performed on tumor tissues of mice using antibodies against IL20RA, p-STAT3 (Tyr705) and CD8α (Santa Cruz Biotechnology, Dallas, TX, USA), SOX2 (Proteintech, Wuhan, Hubei, China) and CD11c (Cell Signaling Technology, Danvers, MA, USA). The images were captured by Olympus BX51 Epifluorescent microscopy (Olympus, Tokyo, Japan).

The expression levels of IL20RA and SOX2 were evaluated according to their staining intensity and the percentage of stained positive cells in each tumor tissue. Their staining intensities were evaluated as negative, weak, moderate, and strong, which were scored as 1, 2, 3, and 4, respectively. For IL20RA, the percentage of positive cells was separated into 0%-25%, 26%-50%, 51%-75%, and 76%-100% subgroups, which were scored as 1, 2, 3, and 4, respectively. For SOX2, the percentage of positive cells was separated into <10%, 10%-29%, 30%-49%, and ≥50% subgroups, which were scored as 1, 2, 3, and 4, respectively. For p-STAT3 (Tyr705), the percentage of positive cells was separated into <5%, 5%-14%, 15%-24%, and ≥25% subgroups, which were scored as 1, 2, 3, and 4, respectively. For CD8α and CD11c, the percentage of positive cells was separated into <5%, 5%-9%, 10%-14%, and ≥15% subgroups, which were scored as 1, 2, 3, and 4, respectively. The cell percentage score and staining intensity score were multiplied to obtain the IHC score.

### Cell culture

Wild-type mouse breast cancer cell lines 4TO7, 4T1, and EO771 and a stable EO771 cell line overexpressing firefly luciferase (EO771FL) were kindly provided by Dr. Ralph A. Reisfeld (The Scripps Research Institute, CA, USA). Mouse breast cancer cell line EMT6 was obtained from the Laboratory Animal Research Center of the Fourth Military Medical University (Xi'an, Shanxi, China). Human breast epithelial cell lines MCF 10A and human breast cancer cell lines MDA-MB-231, HCC1937, MDA-MB-453, MDA-MB-436, T-47D, MCF-7, and ZR-75-1 were obtained from the Cell Bank of the Chinese Academy of Sciences (Shanghai, China); these cell lines were authenticated by short-tandem repeat profiling. The MDA-MB-231 and 4T1 cells were cultured in Dulbecco's Modified Eagle Medium (DMEM) containing 10% fetal bovine serum (FBS), 100 μg/mL streptomycin, and 100 U/mL penicillin. The T-47D cells were maintained in DMEM containing 10% FBS, 1% non-essential amino acids, 100 μg/mL streptomycin and 100 U/mL penicillin. EO771FL cells were maintained in RPMI-1640 medium supplemented with 10% FBS, 100 μg/mL streptomycin, and 100 U/mL penicillin. All cells were cultured in an incubator at 37 °C with 5% CO_2_.

### Cell line establishment

The cDNA of *homo IL20RA* or *mus Il20ra* was inserted into the pLV-EF1α-MCS-IRES-Bsd plasmid. T-47D, 4T1, and EO771FL cells were infected with lentivirus carrying the species-appropriate plasmid. Cells transfected with lentivirus carrying the empty plasmid were used as the control. Cells were selected using blasticidin to obtain the stable polyclonal T-47D, 4T1, and EO771FL cell lines with IL20RA overexpression and their controls (Ctrl). MDA-MB-231 cells were infected with lentivirus carrying pLV-H1-shIL20RA-puro or pLV-H1-shRNA (control)-puro plasmid, then treated with puromycin to obtain the stable polyclonal cell line with *IL20RA* silencing (shIL20RA) and the shRNA control (shCtrl). The sequences of shRNAs were: shIL20RA#1: GCAAACATCACCTTCTTATCC; shIL20RA#2: GGTGGTAAGTTGGTCGCATGT. The sequence of control shRNA was previously described 8.

The cDNA of *mus Il20rb* was inserted into the pLV-EF1α-MCS-IRES-Puro plasmid. EO771FL cells were infected with lentivirus carrying this plasmid. EO771FL cells transfected with lentivirus carrying the empty plasmid were used as the control. Puromycin was used to select stable polyclonal EO771FL cells overexpressing *Il20rb* (EO771FL-IL20RB) and control cells (EO771FL-Ctrl).

### Western blot

Different cell lines were lysed in radioimmunoprecipitation assay (RIPA) buffer containing protease inhibitor cocktail (PIC) and phosphatase inhibitor cocktails 2 and 3 (Sigma-Aldrich, St. Louis, MO, USA). Proteins (20-40 μg) were loaded into 10-12% Tris-Acrylamide gels. The bands were detected using the following primary antibodies: anti-SOX2, OCT4, NANOG, α-tubulin and transforming growth factor beta 1 (TGF-β1) antibodies (Proteintech, Wuhan, Hubei, China); anti-β-actin, IL20RA, p-STAT3 (Tyr705), STAT3, interferon gamma (IFN-γ), IL-2, and tumor necrosis factor alpha (TNF-α) antibodies (Santa Cruz Biotechnology, Dallas, TX, USA); anti-PD-L1 antibody (Novus Biologicals, Centennial, CO, USA); anti-JAK1 and p-JAK1 antibodies (Abcam, Cambridge, UK); and proper horseradish peroxidase-conjugated secondary antibodies.

β-actin or α-tubulin was detected as the loading control. The densitometry of each band was analyzed using ImageJ software (National Institutes of Health, Bethesda, MD, USA), then compared with that of β-actin or α-tubulin of each sample to obtain the normalized relative expression (RE) values for each band.

### Patient information

The study was approved by the Ethics Committee of Nankai University and was performed according to the respective guidelines of the Tianjin Medical University Cancer Institute and Hospital, the Chinese People's Liberation Army (PLA) General Hospital, and the Tianjin Union Medical Centre. The breast tumor samples were collected at the Chinese PLA General Hospital from 2015 to 2018, and their diagnoses were histologically confirmed. Serum was collected from healthy women and female patients with histological evidence of breast cancer who were admitted to the Tianjin Medical University Cancer Institute and Hospital and the Department of Blood Transfusion of the PLA General Hospital from 2014 to 2016. The serum samples were stored at -80 °C.

The fresh human tissue samples, including colorectal normal, para-carcinoma and tumor tissues, were obtained at the Tianjin Union Medical Centre from 2014 to 2019. Their diagnoses were histologically confirmed. These samples were ground using a SCIENTZ-48 high-throughput tissue grinder, then lysed in RIPA buffer supplemented with PIC and phosphatase inhibitor cocktails 2 and 3 (Sigma-Aldrich, St. Louis, MO, USA) and then subjected to western blot analysis.

The patients' clinical information is shown in **Tables S1-S3.**

### Enzyme-linked immunosorbent assay

The ligands of IL20RA (IL-19, IL-20, IL-24, and IL-26) in human serum were quantified using respective enzyme-linked immunosorbent assay (ELISA) kits (Cloud-clone, Wuhan, Hubei, China) according to the manufacturer's instruction. A microplate reader (Promega, Madison, WI, USA) was used to measure the optical density at 450 nm.

### SiRNA transfection

SiRNA transfection assay was performed by the N-TER^TM^ Nanoparticle siRNA Transfection System following the manufacturer's protocol (Sigma-Aldrich, St. Louis, MO, USA). MDA-MB-231 cells were transfected with siRNA Ctrl or mixture of three IL20RA siRNAs, the final concentration of siRNA used was 25 nM. After 48 h of transfection, the cells were harvested and then subjected to the follow-up experiments. The siRNA sequences were summarized in **Table S4**.

### Side population assay

Cells were seeded in 6-well plates and cultured until their density reached 90%. They were washed twice with phosphate-buffered saline (PBS) and cultured in 1 mL pre-warmed staining buffer (PBS supplemented with 2% FBS). The cells were then cultured with 1 μg/mL (for MDA-MB-231), 8 μg/mL (for T-47D and EO771FL), or 7 μg/mL (for 4T1) Hoechst 33342 dye with or without 5 μM reserpine at 37 °C for 60 min, then digested and centrifuged at 1000 rpm at 4 °C for 5 min and re-suspended in 1 mL pre-chilled staining buffer containing 1 μg/mL propidium iodide (PI) for dead cell discrimination. The cells were subjected to flow cytometric analysis using LSRFortessa flow cytometer (BD Biosciences, San Jose, CA, USA). Data were analyzed using FlowJo software (BD Biosciences, San Jose, CA, USA).

### Sphere formation assay

Breast cancer cells were harvested and re-suspended in sphere formation medium, which was composed of RPMI-1640 (for EO771FL) or DMEM (for 4T1, MDA-MB-231, and T-47D) supplemented with 1 × B27, 20 ng/mL fibroblast growth factor-basic and 20 ng/mL epidermal growth factor (Thermo Fisher Scientific, Waltham, MA, USA). Cells were plated in a 48-well Clear Flat Bottom Ultra-Low Attachment Microplate (CORNING, Corning, NY, USA) at a density of 2000, 1000, 500, 250, or 100 cells/well. After 7 days (for EO771FL cells and 4T1 cells) or 10 days (for MDA-MB-231 and T-47D cells), the spheres (defined as >20 cell/spheroid) were recorded using Olympus BX51 Epifluorescent microscopy (Olympus, Tokyo, Japan).

### ALDEFLUOR assay

Aldehyde dehydrogenase (ALDH) assay was performed using the ALDEFLUOR Kit following the manufacturer's protocol (STEMCELL Technologies, Vancouver, Canada). Briefly, 2.5 × 10^5^ cells were suspended in 500 μL ALDEFLUOR assay buffer containing the ALDH substrate and then incubated at 37 °C for 45 min. A specific ALDH inhibitor N, N-Diethylaminobenzaldehyde (DEAB) was used as the control to block ALDH activity. The cell samples were analyzed on a FACSCalibur flow cytometer (BD Biosciences, San Jose, CA, USA), and data were analyzed using FlowJo software (BD Biosciences, San Jose, CA, USA).

### Real-time PCR

TRIZOL (Invitrogen) was used to extract total RNA from cells, reverse transcription was performed using the M-MLV reverse transcriptase (Promega, Madison, WI, USA). Real-time PCR was performed using the Hieff qPCR SYBR® Green Master Mix (YEASEN Biotechnology, Shanghai, China). The 2^-ΔΔCt^ method was used to obtain the relative fold change (RFC) of each mRNA. *GAPDH/Gapdh* was detected as the loading control. The primer sequences are shown in **Table S5**.

### Apoptosis assay

To assess apoptosis, 3 × 10^5^ cells were plated in each well of a 6-well plate and cultured overnight. Cisplatin (DDP), doxorubicin (DOX), or cyclophosphamide (CTX) was then added to the cell culture medium. The concentrations of DDP used were 2 μg/mL for T-47D cells and 8 μg/mL for MDA-MB-231 cells, the concentration of DOX used was 1 μg/mL for 4T1 and EO771FL cells, and the concentration of CTX used was 10 μg/mL for 4T1 and EO771FL cells. The cells were harvested 48 h after treatment and re-suspended in 1× binding buffer (BD Biosciences, San Jose, CA, USA). Cells were stained with Annexin Ⅴ-FITC and PI (BD Biosciences, San Jose, CA, USA) and then subjected to flow cytometric analysis using the FACSCalibur flow cytometer (BD Biosciences, San Jose, CA, USA).

### Preparation of liposomal nanoparticles

Liposomal nanoparticles (NPs) were prepared as described before 40. Briefly, the liposomes were composed of 1,2-dioleoyl-sn-glycero-3-phosphoethanolamine (DOPE), 1,2-dioleoyl-sn-glycero-3-phosphocholine (DOPC), and cholesterol (1:1:1 molar ratio). For preparation of 1 mL of the liposomal NPs encapsulating the STAT3 inhibitor stattic, 1.25 μL stattic (50 mg/mL) was dissolved in 1 mL PBS (pH 7.4) for 15 min. To achieve complete hydration, the mixture was placed into a rotary evaporator for 1 h. To form NPs of an appropriate diameter, the mixture was extruded through a 100 nm polycarbonate membrane filter 10 times using a mini-extruder. To prepare the IL20RA-targeted liposomal NPs, DSPE-PEG3400-NHS was mixed with a monoclonal anti-IL20RA antibody (sc-80065, Santa Cruz Biotechnology) at a mass ratio of 1 mg: 20 μg (micelle: antibody). The IL20RA-PEG conjugates were then mixed with liposomes at a 1:100 molar ratio.

### Animal studies

All animal studies were approved by the Research Institute Ethics Committee of Nankai University and were performed according to the guidelines on the use and care of laboratory animals of Nankai University.

Female NOD/SCID mice at 6-8 weeks of age were separated randomly into two groups. Next, 2 × 10^6^ T-47D-Ctrl or T-47D-IL20RA cells were injected into the fourth mammary fat pad of each mouse. Tumor volume was measured using calipers and calculated using the formula: (length × width^2^)/2. Forty-five days after inoculation, the mice were sacrificed and subjected to lung metastatic analysis. The percentage of lung metastatic area was calculated using the formula: (area of metastatic foci/ total area of five lung lobes) × 100%. The area was measured using Photoshop software (Adobe, San Jose, CA, USA).

Female C57BL/6 (for EO771FL inoculation) and BALB/c (for 4T1 inoculation) mice at 6-8 weeks of age were randomized into different groups, respectively, for the described experiments. For limiting-dilution orthotopic transplantation, 1 × 10^5^, 5 × 10^4^, or 1 × 10^4^ of EO771FL-Ctrl and EO771FL-IL20RA cells and 5 × 10^4^, 1 × 10^4^, or 5 × 10^3^ of 4T1-Ctrl and 4T1-IL20RA cells were injected into the fourth mammary fat pad of each C57BL/6 or BALB/c mouse, respectively. For bioluminescent imaging, 2 × 10^6^ EO771FL cells were injected into each C57BL/6 mouse through the tail vein. Bioluminescent images were obtained following the methods previously described 41. Tumor and lung tissues were collected and subjected to hematoxylin and eosin (H&E) and IHC staining.

To detect the expression of protein in tumor allografts, the tumor allografts were cut into small pieces and ground using a SCIENTZ-48 high-throughput tissue grinder in RIPA lysis buffer supplemented with PIC and phosphatase inhibitor cocktails 2 and 3, then subjected to western blot analysis.

### Doxorubicin and cyclophosphamide treatment *in vivo*

For this experiment, 1 × 10^5^ 4T1-IL20RA or 4T1-Ctrl cells were injected into the fourth mammary fat pad of each female BALB/c mice aged 6-8 weeks. Mice bearing 4T1-IL20RA or 4T1-Ctrl were randomized into two groups, respectively. DOX and CTX treatment began 8 days after inoculation. CTX (50 mg/kg) or PBS was administrated to the mice every other day for a total of 8 times via intraperitoneal injection (i.p.). DOX (5 mg/kg) or PBS was injected once per week for three weeks through the tail vein. Mice were euthanized 40 days after inoculation.

### CDDO-Im treatment *in vivo*

For this experiment, 1 × 10^5^ 4T1-IL20RA cells were injected into the fourth mammary fat pad of female BALB/c mouse aged 6-8 weeks. Tumor-bearing mice were randomized into two groups, and STAT3 inhibitor (CDDO-Im) treatment began 8 days after inoculation. Animals were intratumorally injected with CDDO-Im (2.5 mg/kg) or dimethyl sulfoxide (DMSO) every day for 14 days.

### Analysis of nanoparticle distribution in different organs/tissues of BALB/c mice

For analysis of NP distribution, 1 × 10^5^ 4T1-IL20RA cells were injected into the fourth mammary fat pad of female BALB/c mice aged 6-8 weeks. The tumor-bearing mice were randomized into two groups. Each mouse was injected with 200 μL NPs-DOX or 200 μL NPs-DOX-IL20RA via tail vein on the tenth day after inoculation. The mice were sacrificed 4 h after injection. Various organs/tissues were removed in order to detect the distribution of DOX. The method used to measure the concentration of DOX in the tissues was previously described 40.

### Immunofluorescence staining

The frozen tissue sections from BALB/c mice treated with NPs-DOX or NPs-DOX-IL20RA were counterstained with DAPI. The slices were then subjected to confocal imaging (Olympus, Tokyo, Japan).

### Flow cytometric analysis of tumor-infiltrating lymphocytes

5 × 10^4^ 4T1-IL20RA or 4T1-Ctrl cells were injected into the fourth mammary fat pad of each female BALB/c mouse aged 6-8 weeks, while 7.5 × 10^5^ EO771FL-IL20RA or EO771FL-Ctrl cells were injected into the fourth mammary fat pad of each female C57BL/6 mouse aged 6-8 weeks. Twenty-five days after inoculation, the mice were sacrificed. Primary tumor tissues were removed and single-cell suspensions were obtained as previously described 39. Cells from each tissue sample were re-suspended in PBS supplemented with 2% FBS, then separated into three tubes. The first tube contained anti-CD45 Ab-PerCP-Cy5.5, anti-CD4 Ab-APC (BD Biosciences, San Jose, CA, USA), and anti-CD8 Ab-PE-Cy7 (Thermo Fisher Scientific, Waltham, MA, USA); the second tube contained anti-CD45 Ab-PerCP-Cy5.5, anti-CD11b Ab-FITC and anti-Gr1 Ab-PE (BD Biosciences, San Jose, CA, USA); the third tube contained anti-CD45 Ab-FITC (BD Biosciences, San Jose, CA, USA) combined with anti-CD49b Ab-PE (Thermo Fisher Scientific, Waltham, MA, USA) for 4T1 allograft or anti-NK1.1 Ab-APC (Thermo Fisher Scientific, Waltham, MA, USA) for EO771FL allograft. The cells in the tubes were then subjected to flow cytometric analysis.

### Combined therapy experiments

5 × 10^4^ 4T1-IL20RA cells were injected into the fourth mammary fat pad of BALB/c mice. The 4T1-IL20RA allograft mice were randomly separated into five groups. Mice in each group were treated with the following agents, respectively: 1) IgG (Santa Cruz Biotechnology, 100 μg, i.p. every 3 days for four times); 2) anti-PD-L1 antibody (αPD-L1 Ab, BioXCell, Clone 10F.9G2, 100 μg, i.p. every 3 days for four times); 3) NPs-Stattic (200 µL/dose, intravenous injection (i.v.) every 6 days for three times); 4) NPs-Stattic-IL20RA (200 µL/dose, i.v. every 6 days for three times) plus IgG (100 μg, i.p. every 3 days for four times); and 5) NPs-Stattic-IL20RA (200 µL/dose, i.v. every 6 days for three times) plus αPD-L1 Ab (100 μg, i.p. every 3 days for four times). All of the mice were treated with CTX (60 mg/kg) and DOX (2 mg/kg) i.p. every 6 days for three times.

### Bioinformatic analysis

The gene expression profiles of IL20RA in GSE62598 and GSE1323 were acquired from the GEO database (https://www.ncbi.nlm.nih.gov/geoprofiles/). For query of the IL20RA ligands in a breast cancer dataset, 996 breast invasive carcinomas from The Cancer Genome Atlas (TCGA; PanCancer Atlas; obtained from https://www.cbioportal.org/) were analyzed. The TCGA breast cancer dataset which contains raw expression counts of 1102 individual human breast cancer tissues was obtained from the UCSC Xena browser (http://xena.ucsc.edu/). The human breast cancer samples were divided into IL20RA^high^ (n = 551) and IL20RA^low^ (n = 551) groups for gene set enrichment analysis (GSEA). GSEA was performed according to authors' guidelines (http://www.broadinstitute.org/gsea/index.jsp).

### Statistical analysis

Data were expressed as means + standard error of mean (SEM), and statistical significance was determined by *t*-test unless otherwise specified. A *p*-value of <0.05 was used as the criterion for statistical significance.

## Results

### IL20RA is highly expressed in human breast and colorectal cancers and is associated with the expression of SOX2

**Figure 1:**
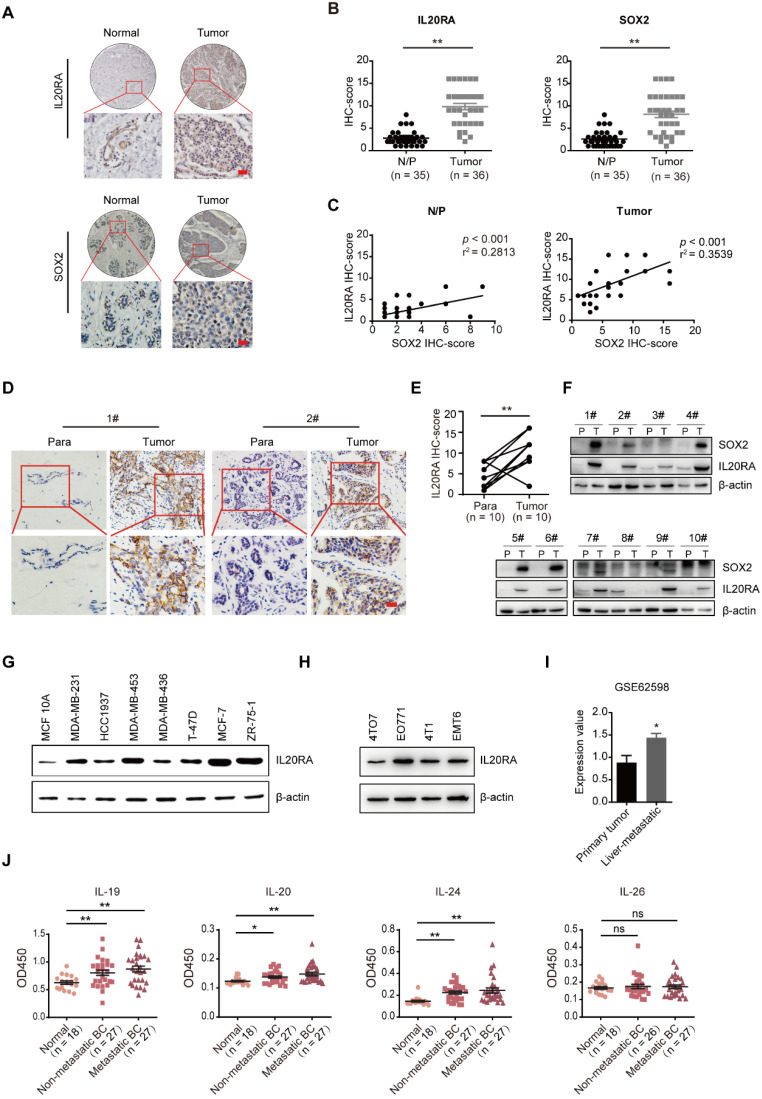
** IL20RA is highly expressed in human breast cancers and correlated with the expression of SOX2. (A)** IHC staining images of IL20RA and SOX2 in breast tissue microarray. Scale bar, 20 µm. **(B)** Statistical analyses of the IHC-scores of IL20RA and SOX2 in breast tissue microarray. N/P: normal/para-carcinoma**.** Data are presented as mean ± SEM.** (C)** Pearson's analyses of the correlation between IL20RA and SOX2 in normal/para-carcinoma and tumor tissues (n = 35 for N/P, n = 36 for tumor). **(D)** Representative IHC staining images of IL20RA in para-carcinoma (Para) and tumor tissues from human breast cancer patients. Scale bar, 20 µm. **(E)** Statistical analysis of the IHC-score of IL20RA in breast cancer and matched para-carcinoma tissues of 10 human breast cancer patients (n = 10 for each group). **(F)** Western blot results of IL20RA and SOX2 in para-carcinoma (P) and tumor (T) tissues of 10 human breast cancer patients. **(G)** Western blot results of IL20RA in human breast epithelial cell line MCF 10A and breast cancer cell lines. **(H)** Western blot results of IL20RA in mouse breast cancer cell lines. **(I)** The expression of *Il20ra* in primary tumor explants and liver-aggressive explants of 4T1 from GEO dataset (GSE62598; n = 3 for each group). **(J)** Statistical analyses for the concentration of IL-19, IL-20, IL-24 and IL-26 in the serum of normal female donors, non-metastatic female breast cancer (BC) patients, metastatic female BC patients. Data are presented as mean ± SEM. *, *p* < 0.05; **, *p* < 0.01; ns, not significant.

To determine whether IL20RA plays a role in breast carcinomas and to further investigate its function in stemness regulation, we examined the expression of IL20RA and SOX2 using tissue microarrays containing human normal/para-carcinoma breast tissues and breast tumors. As shown in **Figure 1A**-**B**, IL20RA and SOX2 were increased in tumor tissues compared to normal/para-carcinoma tissues. The expression of IL20RA was positively correlated with that of SOX2 (**Figure 1C**). In addition, 10 clinical human breast cancer samples (**Figure 1D**) and 24 human colorectal cancer samples (**Figure S1A**) were collected. Elevated expressions of IL20RA and SOX2 in breast tumor tissues were detected when compared to the matched para-carcinoma tissues (**Figure 1D-F**). Higher expression of IL20RA and SOX2 was also found in colorectal cancer tissues compared with matched para-carcinoma or normal tissues (**Figure S1A-B**). A positive correlation was also detected between IL20RA and SOX2 in these colorectal tissues (**Figure S1C**). The relatively higher expression of IL20RA was also found in the human breast cancer cell lines, including MDA-MB-231, MDA-MB-453, T-47D, MCF-7, and ZR-75-1, compared with the non-tumorigenic breast epithelial cell line MCF 10A (**Figure 1G**). The expression of IL20RA was also detectable in four mouse breast cancer cell lines: 4TO7, EO771, 4T1, and EMT6 (**Figure 1H**). Analysis of the public microarray dataset GSE62598 revealed higher expression of *Il20ra* in 4T1 liver-aggressive explants compared with 4T1 primary tumor explants (**Figure 1I**). Analysis of GSE1323 revealed higher expression of *IL20RA* in the cell line derived from corresponding metastasis than in that derived from primary colon tumor (**Figure S1D**).

The ligands of IL20RA were queried using data from TCGA (**Figure S1E**). Amplification of *IL19*,* IL20,* and *IL24* co-occurs in ~9% of breast cancer patients, while the rate of* IL26* amplification is only 2.4%. We also detected the concentration of the ligands in the serum of healthy female donors and female patients with non-metastatic or metastatic breast cancer. We found that the concentrations of IL-19, IL-20, and IL-24, but not IL-26, were increased in the serum of both patients with non-metastatic and metastatic breast cancer compared with that of healthy donors **(Figure 1J)**. These findings indicate that IL20RA signaling may play a role in cancer progression and stemness regulation.

### IL20RA plays a vital role in promoting the stemness of breast cancer *in vitro*

**Figure 2:**
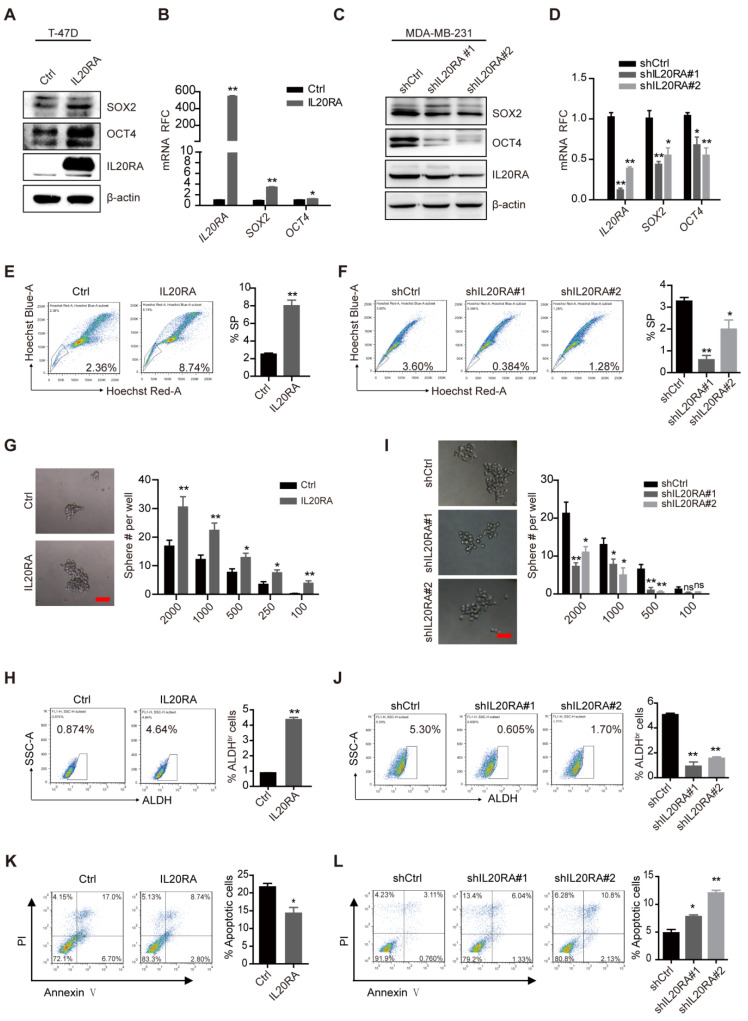
** IL20RA promotes the stemness of breast cancer cells *in vitro*. (A)** Western blot results of SOX2, OCT4, and IL20RA in T-47D cells. **(B)** Real-time PCR analysis of *IL20RA, SOX2,* and *OCT4* mRNAs in T-47D cells (n = 3 for each group). **(C)** Western blot results of SOX2, OCT4, and IL20RA in MDA-MB-231 cells. **(D)** Real-time PCR analysis of *IL20RA*,* SOX2* and* OCT4* mRNAs in MDA-MB-231 cells (n = 3 for each group).** (E**-**F)** Flow cytometric analysis of side population (SP) in T-47D (E) and MDA-MB-231 cells (F). Left panel: representative flow cytometric analysis results, right panel: the statistical analysis of SP proportions (n = 3 for each group).** (G**, **I)** Sphere formation assay in T-47D (G) and MDA-MB-231 cells (I). Left panel: representative images of tumor spheres. Right panel: statistical analysis of sphere number (#; n = 6 for each group of T-47D cells, n = 4 for each group of MDA-MB-231 cells). Scale bar, 50 µm. **(H**, **J)** Flow cytometric analysis of ALDH^br^ population in T-47D (H) and MDA-MB-231 cells (J). Left panel: representative flow cytometric analysis results, right panel: statistical analysis of the proportion of ALDH^br^ populations (n = 3 for each group). **(K**-**L)** Flow cytometric analysis of apoptotic cells in T-47D (K) and MDA-MB-231 cells (L) treated with cisplatin for 48 h. Left panel: representative flow cytometric analysis results, right panel: statistical analysis of the percentage of apoptotic cells (n = 3 for each group). *, *p* < 0.05; **, *p* < 0.01; ns, not significant.

To elucidate the function of IL20RA in breast cancer cell stemness, MDA-MB-231 cells were transiently transfected with *IL20RA* siRNA or siRNA control. Silencing of *IL20RA* reduced the expression of SOX2 and the percentage of side population (SP) cells (**Figure S2A-B**). To further investigate the function of IL20RA, we established stable T-47D cell lines overexpressing *IL20RA* and MDA-MB-231 cell lines with downregulation of *IL20RA*. Consistently,* IL20RA* overexpression increased the expression of stemness marker genes, such as *SOX2* and *OCT4*, in T-47D cells (**Figure 2A-B**), while knockdown *IL20RA* decreased the expression of these genes in MDA-MB-231 cells (**Figure 2C-D**). In addition, *IL20RA* overexpression significantly increased the proportion of SP cells (**Figure 2E**), while silencing of *IL20RA* showed the opposite effect (**Figure 2F**). Overexpression of *IL20RA* also promoted sphere formation ability (**Figure 2G**) and enhanced the ALDH^br^ population of T-47D cells (**Figure 2H**), while silencing of *IL20RA* showed inhibitory effects on the sphere-formation ability and ALDH^br^ population of MDA-MB-231 cells (**Figure 2I-J**). In addition, expression of *Sox2/SOX2* and *Il20ra/IL20RA* was enriched in the sorted SP and/or tumor spheres of EO771FL and T-47D cells (**Figure S2C-D**). The sphere-enriched expression of IL20RA was further verified in MCF-7, MDA-MB-231, EMT6, and 4TO7 cells (**Figure S2E**). These findings provide evidence for the stemness-promoting effects of IL20RA signaling in breast cancer cells.

Increased chemoresistance is another feature of stemness. We found that overexpression of *IL20RA* inhibited, but knockdown of *IL20RA* enhanced, DDP-induced apoptosis in T-47D and MDA-MB-231 cells, respectively (**Figure 2K-L**). These results further support the stemness-promoting effect of IL20RA on cancer cells.

### IL20RA signaling promotes the stemness features of breast cancer *in vivo*

**Figure 3:**
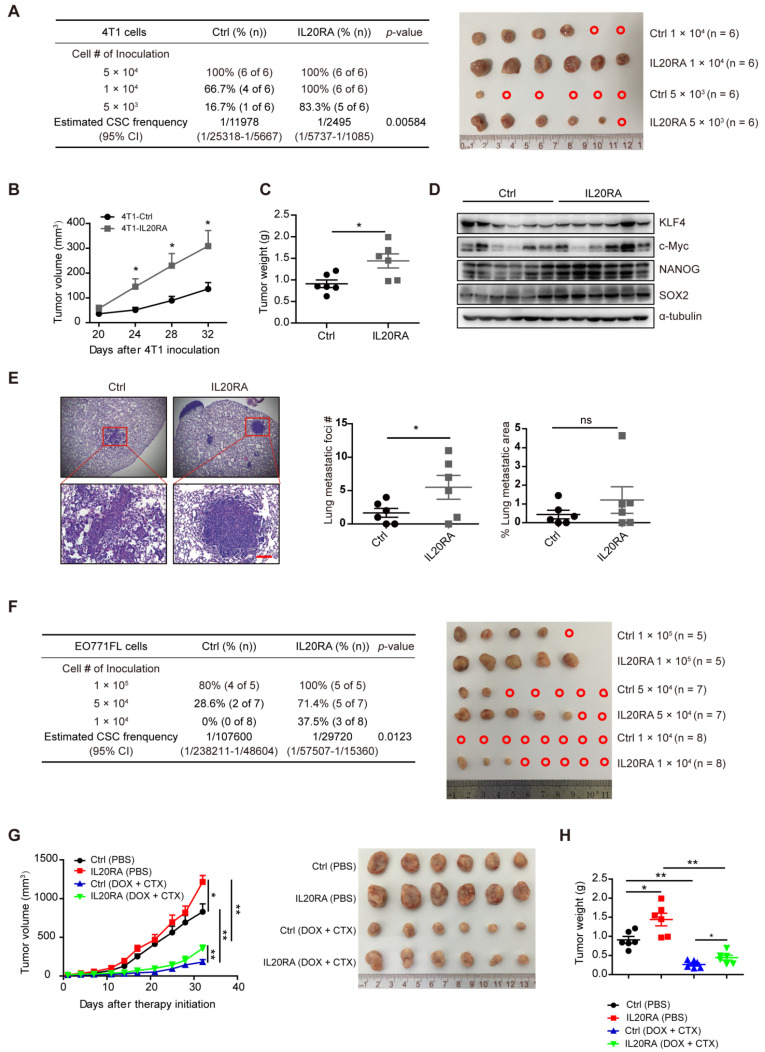
** IL20RA signaling promotes the stemness features of breast cancer *in vivo*. (A)** Left panel: ELDA analysis of the tumor-initiating ability of 4T1 cells, which were injected into the 4th mammary fat pad of BALB/c mice. Right panel: primary tumors derived from different groups. A red open circle indicates no tumor formation. (**B**-**E**) The data of tumor growth curve, tumor weight, western blot and lung metastasis come from the 5 × 10^4^ cell-inoculation group: **(B)** Tumor growth curves of the 4T1-allograft mice (n = 6 mice for each group). **(C)** Statistical analysis of tumor weights at the end of experiments. Data are presented as mean ± SEM (n = 6 mice for each group). **(D)** Western blot of KLF4, c-Myc, NANOG and SOX2 in 4T1 allografts. **(E)** Left panel: representative H&E staining images of lung metastasis in 4T1-allograft mouse models. Scale bar, 100 µm. Right panel: statistical analyses of the metastatic foci number and the percentage of metastatic area of lungs. Data are presented as mean ± SEM, n = 6 mice for each group.** (F)** Left panel: ELDA analysis of the tumor-initiating ability of EO771FL cells, which were injected into the 4th mammary fat pad of C57BL/6 mice. Right panel: primary tumors derived from different groups. A red open circle indicates no tumor formation.** (G)** Left panel: tumor growth curves of 4T1-allograft mice receiving DOX plus CTX combined treatment or PBS treatment (n = 6 mice for each group). Tumor volumes between different groups are compared 32 days after the initiation of therapy to determine the statistical significances. Right panel: primary tumors separated from different groups at the end of the experiments. **(H)** Statistical analysis of tumor weights at the end of experiments. Data are presented as mean ± SEM, n = 6 mice for each group. *, *p* < 0.05; **, *p* < 0.01; ns, not significant. Abbreviation: CI, confidence interval.

To examine the stemness-promoting effects of IL20RA *in vivo*, we constructed stable 4T1 and EO771FL cell lines overexpressing *Il20ra* (4T1-IL20RA, EO771FL-IL20RA). *Il20ra* overexpression significantly increased the expression of stemness marker genes *Sox2* and* Oct4* (**Figure S3A-B**). It also increased the proportion of SP cells and promoted the sphere formation ability of 4T1 and EO771FL cells (**Figure S3C-F**). To further verify the stemness-promoting effects of IL20RA signaling *in vivo*, an orthotopic allograft model was established. For this model, 4T1-IL20RA and control (4T1-Ctrl) cells were injected into the fourth mammary fat pad of female BALB/c mice. The frequency of CSCs was determined using extreme limiting dilution analysis (ELDA) software. Our results revealed an increased frequency of CSCs in the 4T1-IL20RA inoculation group compared to the 4T1-Ctrl inoculation group (**Figure 3A**). Increases in tumor volume and tumor weight in the 4T1-IL20RA inoculation group compared with the 4T1-Ctrl inoculation group were also observed (**Figure 3B-C**). We found that IL20RA overexpression boosted the expression of the stemness marker genes* Nanog* and* Sox2* in tumor tissues *in vivo* (**Figure 3D**) and increased the number of lung metastatic foci (**Figure 3E**). These results demonstrated the ability of IL20RA signaling to promote stemness features in breast cancer *in vivo*. This conclusion was further supported by another allograft model, in which female C57BL/6 mice were inoculated with EO771FL-IL20RA and control cells (EO771FL-Ctrl). ELDA revealed an increased frequency of CSCs in the EO771FL-IL20RA inoculation group compared to the control group **(Figure 3F)**.

Flow cytometric analysis showed that IL20RA also enhanced the resistance of 4T1 and EO771FL cells to DOX and CTX (**Figure S3G-H**). To further test the chemoresistance-promoting effects of IL20RA *in vivo*, a 4T1 allograft mouse model was established. We found that, although DOX and CTX significantly inhibited the tumor growth and tumor weight in both groups (**Figure 3G-H**), the volume of 4T1-IL20RA allografts increased faster than the control group after treatment (**Figure 3G**). At the same time, the body weight of mice was monitored and no significant difference between the 4T1-IL20RA and respective 4T1-Ctrl inoculation groups with or without chemotherapy was observed (**Figure S3I**). Taken together, these findings support the stemness-promoting effects of IL20RA* in vivo*.

As IL20RB is a functional partner of IL20RA, its effects on the stemness properties of breast cancer cells were also investigated. We established a stable EO771FL cell line with overexpression of *Il20rb* (EO771FL-IL20RB) and its control (EO771FL-Ctrl;**Figure S4A-B**). We found that overexpression of *Il20rb* in EO771FL cells did not change the expression of core stemness genes, such as *Sox2*, *Oct4*, and *Nanog* (**Figure S4B-C**), nor did it affect the percentage of SP cells (**Figure S4D**). Although IL20RB increased the sphere formation ability at a relatively low cell density (10^3^/well, **Figure S4E**), it had no influence on tumor-initiation ability, as measured by ELDA *in vivo* (**Figure S4F**)*.* The expression of OCT4 and SOX2 did not change in EO771FL-IL20RB allografts compared to EO771FL-Ctrl allografts (**Figure S4G**). However, the lung metastatic burden significantly increased in the mice bearing EO771FL-IL20RB allografts (**Figure S4H**). The metastasis-promoting effect of IL20RB was also observed in the tail-vein injection mouse model, in which the lung metastases of EO771FL cells were traced via bioluminescent imaging (BLI; **Figure S4I**). An increased number of lung metastatic foci was also observed in the EO771FL-IL20RB inoculation group compared to the EO771FL-Ctrl inoculation group at the end of the experiment (**Figure S4J**).

### IL20RA promotes breast cancer stemness via JAK1-STAT3-SOX2 signaling

**Figure 4:**
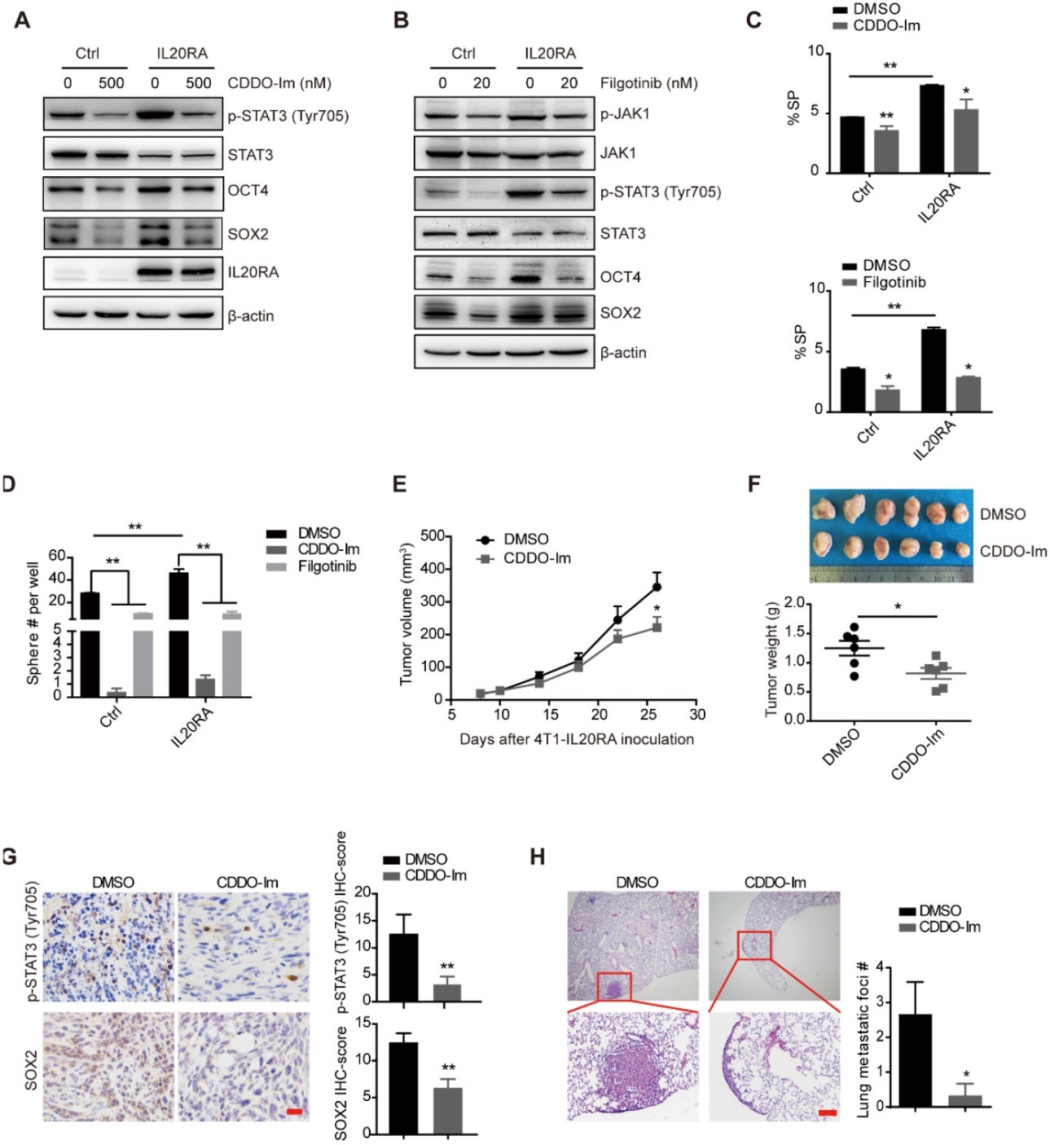
** IL20RA promotes breast cancer stemness via STAT3 activation**. **(A-B)** Western blot results of p-STAT3(Tyr705), STAT3, p-JAK1, JAK1, OCT4, SOX2 and IL20RA in T-47D cells treated with STAT3 inhibitor (CDDO-Im) or JAK1 inhibitor (filgotinib) for 48 h. **(C)** Statistical analysis of the proportion of SP in T-47D cells treated with CDDO-Im and filgotinib, respectively (n = 3 for each group).** (D)** Statistical analysis of the sphere-formation in T-47D cells, which were treated with CDDO-Im and filgotinib, respectively (n = 3 for each group).** (E**-**H)** BALB/c mice bearing 4T1-IL20RA allografts were treated with DMSO and CDDO-Im, respectively. **(E)** Tumor growth curves (n = 6 for each group). **(F)** Upper panel: primary tumors separated from each group at the end of experiments. Lower panel: statistical analysis of tumor weights. Data are presented as mean ± SEM (n = 6 for each group). **(G)** Left panel: representative IHC staining images of p-STAT3 (Tyr705) and SOX2 in 4T1-IL20RA allograft tissues. Scale bar, 20 μm. Right panel: statistical analyses of the IHC-scores of p-STAT3 (Tyr705) and SOX2 (n=6 for each group) in paraffin-embedded tumor tissue sections. **(H)** Left panel: representative H&E staining images of lung metastasis in 4T1-IL20RA allograft mice, scale bar, 100 µm. Right panel: statistical analysis of the lung metastatic foci number (n = 6 for each group). *, *p* < 0.05; **, *p* < 0.01.

Previous studies revealed the activation of JAK signaling pathway upon IL20RA activation 12. We found that overexpression of *IL20RA* increased the expression levels of p-STAT3 (Tyr705) and p-JAK1 (**Figure 4A-B**). Inhibition of STAT3 activation using the small-molecule inhibitor CDDO-Im and filgotinib, a JAK1 inhibitor, effectively blocked the upregulation of stemness marker genes such as *OCT4* and *SOX2* in T-47D-IL20RA cells (**Figure 4A-B**). In addition, the increased SP proportion and sphere formation ability induced by *IL20RA* overexpression were compromised after CDDO-Im and filgotinib treatment in T-47D cells (**Figure 4C-D**). Administration of CDDO-Im suppressed tumor volume and tumor weight of 4T1-IL20RA allografts (**Figure 4E-F**). IHC staining showed that CDDO-Im attenuated the expression of p-STAT3 (Tyr705) and SOX2 and reduced the number of metastatic foci in the lungs (**Figure 4G-H**).

To further verify our findings* in vivo*, we injected T-47D-Ctrl and T-47D-IL20RA cells into NOD/SCID mice. Although there was no significant difference in tumor growth between these two groups (**Figure S5A**), a promotion of lung metastasis occurred in the T-47D-IL20RA inoculation group compared with the T-47D-Ctrl inoculation group (**Figure S5B**). We also found increased expression of p-STAT3 (Tyr705) and SOX2 in the primary tumors of the T-47D-IL20RA inoculation mice via IHC and western blot (**Figure S5C-D**). The activation of STAT3 signaling was also found in the 4T1-IL20RA and EO771FL-IL20RA allografts *in vivo* (**Figure S5E-F**). STAT3 signaling was found to be an important pathway involved in the transcriptional regulation of *Sox2* to promote the stemness of cancer cells 11. These findings suggested that IL20RA activated JAK1-STAT3 to promote the stemness of breast cancer cells and that targeting IL20RA^+^ cells may be a promising strategy to reduce the stemness properties and decrease the chemoresistance of breast cancer cells.

### IL20RA promotes the formation of an immunosuppressive microenvironment in breast cancer

**Figure 5:**
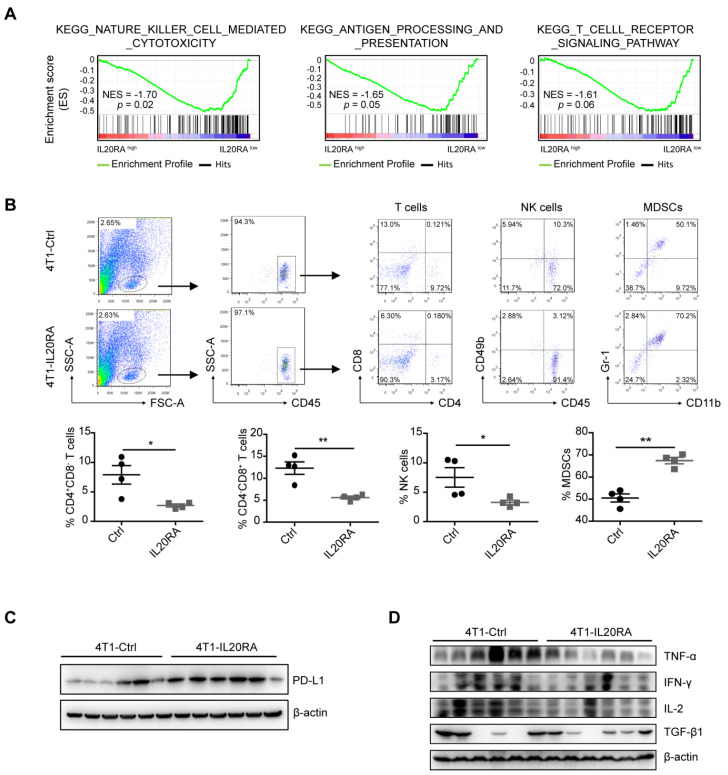
** IL20RA promotes the formation of an immunosuppressive microenvironment in breast cancer. (A)** GSEA analysis of TCGA dataset of breast cancer patients. **(B)** Flow cytometric analysis of the proportion of TILs in 4T1-allografts of BALB/c mice. Cell populations were identified as CD4^+^ T cells (CD45^+^CD4^+^CD8^-^), CD8^+^ T cells (CD45^+^CD4^-^CD8^+^), NK cells (CD45^+^CD49b^+^), MDSCs (CD45^+^CD11b^+^Gr1^+^). Upper panel: representative flow cytometric analysis results. Lower panel: statistical analyses of the proportion of TILs in CD45^+^ cells. Data are presented as mean ± SEM. n = 4 mice for each group. **(C)** Western blot results of PD-L1 in 4T1-allograft tissues.** (D)** Western blot results of TNF-α, IFN-γ, IL-2 and TGF-β1 in 4T1-allograft tissues, *, *p* <0.05; **, *p* < 0.01. Abbreviation: NES, normalized enrichment score.

CSCs possess intrinsic biological characteristics to evade immune surveillance during tumor progression 42. Overexpression of IL20RA had no effect on tumor growth in NOD/SCID mice, whereas IL20RA-overexpressing 4T1 tumors grew markedly faster in immunocompetent mice compared with the 4T1-Ctrl allograft. These observations suggest that IL20RA might also regulate the immune microenvironment of primary breast cancer to promote its progression. To assess the potential link between IL20RA and immune cell infiltration in human breast cancer, the breast cancer database of TCGA was analyzed. GSEA revealed that NK cell-mediated cytotoxicity, antigen processing and presentation, and T-cell receptor signaling pathway were enriched or tended to be enriched in the IL20RA^low^ group, suggesting the existence of an immune-active status in the IL20RA^low^ population (**Figure 5A**). Based on this finding, we hypothesized that IL20RA might promote the formation of an immune environment favorable to cancer cells. Thus, we compared infiltration of immune cells in tumor allografts by flow cytometric analysis. Decreases in CD4^+^ and CD8^+^ T cells and NK cells and an increase in myeloid-derived suppressor cells (MDSCs) were found in 4T1-IL20RA allografts compared with 4T1-Ctrl allografts (**Figures 5B**)**.** These changes in immune cell infiltration were also observed in EO771FL-IL20RA allografts compared with EO771FL-Ctrl allografts (**Figure S6A**).

The PD-1/PD-L1 immune checkpoint plays a pivotal immunosuppressive role in multiple cancers, including breast cancer 43. We found increased expression of PD-L1 in both 4T1-IL20RA and EO771FL-IL20RA allografts tissues compared with their respective controls (**Figures 5C, and S6B**). We found that overexpression of IL20RA also increased the expression of *Cd274/CD274* in 4T1, EO771FL, and T-47D cells (**Figures S6C-D**). The increased expression of PD-L1 could be inhibited by CDDO-Im (**Figure S6D**). In addition, the production of TNF-α, IFN-γ, and IL-2 was reduced in both 4T1-IL20RA and EO771FL-IL20RA allograft tissues compared with their respective controls (**Figure 5D, S6E**). However, the expression and secretion of IFN-γ, TNF-α, and IL-2 were not changed in 4T1-IL20RA cell line compared to 4T1-Ctrl cell line (**Figure S6F**). Although the secretion of TGF-β1 by 4T1-IL20RA cell line was decreased, its expression in 4T1-IL20RA cell line and allografts were not markedly changed compared with the respective 4T1-Ctrl cell line and allografts. These data suggest that the changes in these cytokines were mainly due to the alterations of tumor-infiltrating lymphocytes (TILs) in 4T1-IL20RA allografts. These findings indicate a potential role of the IL20RA-PD-L1 axis in shaping the immune microenvironment of breast cancer by reducing the enrichment of anti-cancer CD8^+^ T lymphocytes and NK cells and decreasing the production of cytokines by TILs. These factors may promote the formation of a tumor-favorable environment.

### Combined with anti-PD-L1 antibody and IL20RA-targeted nanoparticles, the chemotherapeutic efficacy in a breast cancer model with high IL20RA expression is improved

**Figure 6:**
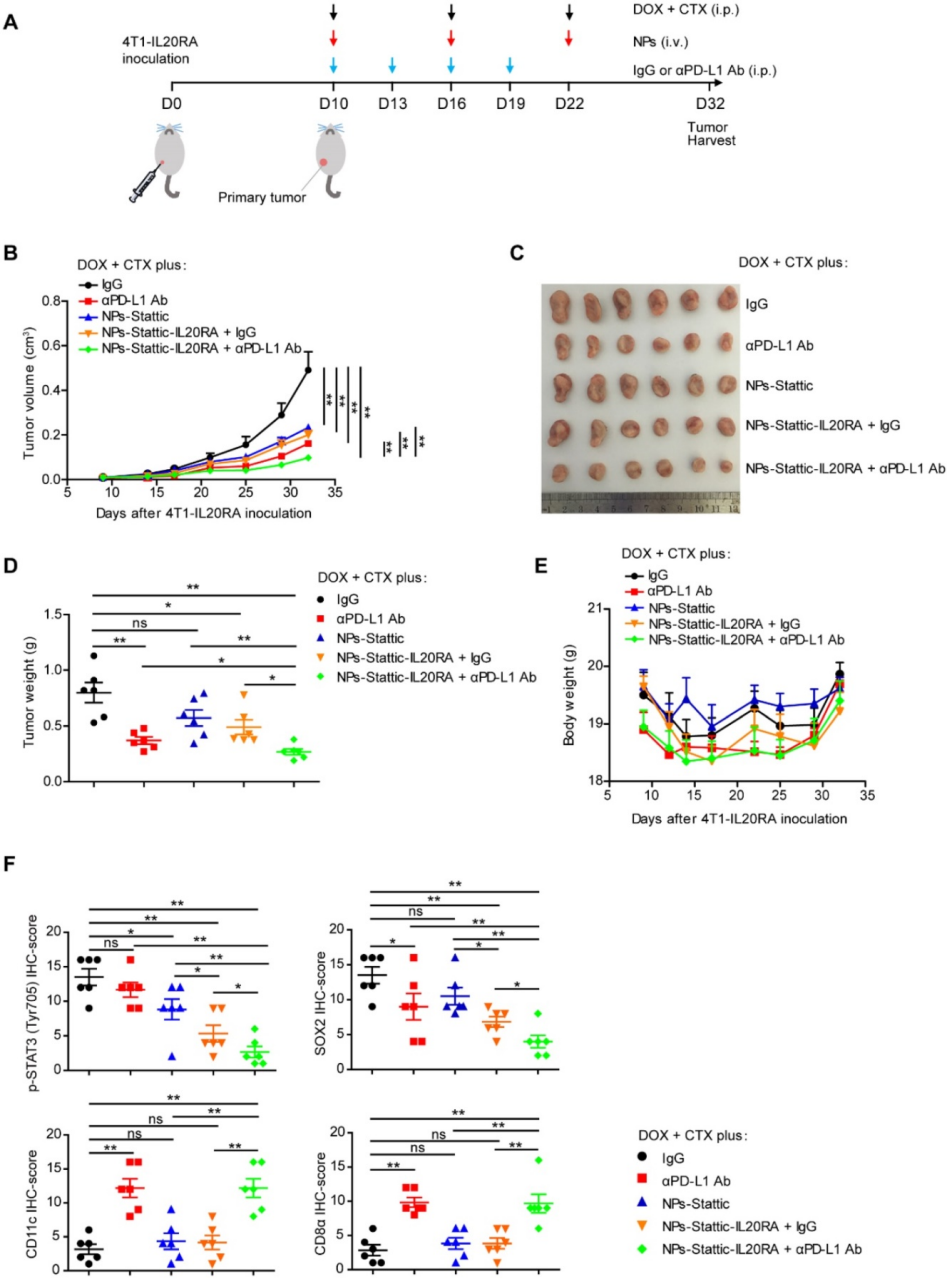
** Combination of immunotherapy and targeted therapy with chemotherapy for breast cancer with high IL20RA expression. (A)** Schematic diagram of the experimental procedure. **(B-D)** BALB/c mice bearing 4T1-IL20RA allografts were treated with DOX plus CTX combined with IgG, αPD-L1 Ab, NPs-Stattic, NPs-Stattic-IL20RA plus IgG, and NPs-Stattic-IL20RA plus αPD-L1 Ab, respectively. **(B)** Tumor growth curves (n = 6 mice for each group). Tumor volumes between different groups are compared 32 days after inoculation to determine the statistical significances. **(C)** The image of primary tumors separated from different groups at the end of experiments. **(D)** Statistical analysis of tumor weights at the end of experiments. Data are presented as mean ± SEM, n = 6 mice for each group. **(E)** Body weight curves of BALB/c mice from different groups (n = 6 mice for each group). **(F)** Statistical analyses of the IHC-scores of p-STAT3 (Tyr705), SOX2, CD11c and CD8α in paraffin-embedded tumor tissue sections. Data are presented as mean ± SEM, n = 6 mice for each group. *, *p* < 0.05; **, *p* < 0.01; ns, not significant.

Since we found that IL20RA promotes the stemness of breast cancer cells via the JAK1-STAT3-SOX2 signaling pathway and regulates the expression of PD-L1 to modulate the immune microenvironment, we next asked whether combination of an anti-PD-L1 antibody and IL20RA-targeted delivery of a STAT3 inhibitor could increase the efficacy of traditional chemotherapy. To test this hypothesis, we constructed a novel IL20RA-targeted liposomal NP carrying the STAT3 inhibitor stattic (NP-Stattic-IL20RA) to target IL20RA^+^ tumor cells. Stattic and anti-IL20RA Ab all inhibited the activation of STAT3 in a dose-dependent manner (**Figure S7A-B**). The mean diameter of NPs-Stattic-IL20RA was ~120 nm (**Figure S8A-B**). To test the efficacy of IL20RA-targeted liposomes *in vivo*, we injected IL20RA-targeted nanoparticles encapsulating DOX (NPs-DOX-IL20RA) via tail vein into BALB/c mice bearing 4T1-IL20RA allografts. Compared with the control group, the IL20RA-targeted NPs group exhibited a ~2-fold higher accumulation of DOX in tumor tissues, while the nonspecific accumulation of DOX in the non-targeted organs such as liver, spleen, kidney, and intestine was reduced (**Figure S8C-D**). These results revealed that our liposomal NPs could specifically deliver these agents to cancer tissues. Subsequently, IgG, anti-PD-L1 antibody (αPD-L1 Ab), NPs-Stattic, NPs-Stattic-IL20RA plus IgG, or NPs-Stattic-IL20RA plus αPD-L1 Ab were combined with DOX and CTX and administered to BALB/c mice bearing 4T1-IL20RA allografts (**Figure 6A**). We found that, compared with the IgG control, these four therapeutic strategies all decreased tumor volume and thus boosted the therapeutic effect of DOX and CTX. Most importantly, αPD-L1 Ab combined with NPs-Stattic-IL20RA significantly inhibited the tumor volume and tumor weight of 4T1-IL20RA allografts compared with IgG, αPD-L1 Ab, NPs-Stattic, and NP-Stattic-IL20RA plus IgG treatment groups, respectively (**Figure 6B-D**). Compared to the IgG control, there were no significant differences in the body weights of mice receiving αPD-L1 Ab, NPs-Stattic, NPs-Stattic-IL20RA plus IgG, or NPs-Stattic-IL20RA plus αPD-L1 Ab treatment at the beginning and end of the treatment (**Figure 6E**). IHC staining revealed that αPD-L1 Ab in combination with NPs-Stattic-IL20RA inhibited the expression of p-STAT3 (Tyr705) and SOX2 in tumor tissues compared with IgG, αPD-L1 Ab, NPs-Stattic, and NP-Stattic-IL20RA plus IgG treatment, respectively (**Figure 6F**). In addition, this combination treatment enhanced the expression of CD8α and CD11c in tumor tissues compared with IgG, NPs-Stattic, and NP-Stattic-IL20RA plus IgG treatment, respectively (**Figure 6F**). These results indicate that NPs-Stattic-IL20RA in combination with αPD-L1 Ab can effectively increase the therapeutic effects of DOX and CTX. Furthermore, this therapeutic strategy inhibits the stemness and modifies the immune microenvironment of breast cancer and thus may be a promising therapeutic strategy for breast cancer.

## Discussion

Current studies revealed that CSCs play an important role in the chemoresistance, recurrence and metastasis of various types of cancer. Therefore, finding new signaling pathways that regulate the stemness of cancer cells will form the basis for developing new therapeutic strategies to target CSCs and improve the therapeutic efficacy of cancer.

In this study, we found that IL20RA expression was elevated in breast cancer and colorectal cancer. The expression of IL20RA was positively correlated with that of SOX2 in tumors and noncancerous tissues of both breast cancer and colorectal cancer patients. We also found that the expression level of IL20RA ligands IL-19, IL-20, and IL-24 was dramatically elevated in the serum of breast cancer patients compared to that of healthy donors, indicating that IL20RA signaling may play an important role in the progression of cancer.

We found that IL20RA signaling promoted various stemness properties of breast cancer cells, including higher ALDH activity, increased sphere formation ability, and increased proportion of SP cells, and enhanced resistance to chemotherapeutic agents* in vitro*. IL20RA also promoted the tumor-initiating capacity of breast cancer cells and tumor growth *in vivo*. Furthermore, IL20RA overexpression increased the lung metastasis of breast cancer *in vivo*.

In addition, we found that the JAK1-STAT3 signaling pathway played an important role in mediating the stemness-promoting function of IL20RA signaling and revealed that IL20RA-JAK1-STAT3 signaling promoted the expression of core stemness genes, including *SOX2* and *OCT4*. Furthermore, we discovered that IL20RA could regulate the expression of PD-L1 both *in vitro* and *in vivo*. Activation of STAT3 was reported to be strongly associated with the expression of PD-L1 in multiple hematological malignancies, including lymphoma and myeloproliferative neoplasms 44, 45. We demonstrated that inhibition of STAT3 resulted in reduced PD-L1 expression in breast cancer cells. Most importantly, our study highlighted a prominent role of IL20RA in driving a cancer-favorable immune environment via reduction of CD8^+^ T-cell, and NK-cell infiltration. The mechanism by which IL20RA regulates the recruitment of immune cells to the tumor microenvironment still needs to be further investigated.

Immunotherapy has emerged as a new therapeutic method for breast cancer. Many new immune checkpoint agents have been approved by the United States Food and Drug Administration for the treatment of different cancer types. However, the clinical efficacy of anti-PD-L1 or anti-PD-1 antibody is moderate in metastatic breast cancer 46. DOX and CTX are frequently used in the treatment of breast cancer, either as monotherapy or in combination with other agents 47. Previous reports demonstrated that DOX and CTX treatment could induce the expansion of monocytic myeloid cells, which possess immunosuppressive activities 48, 49. Thus, the combination of immune checkpoint inhibitors with targeted therapy and traditional chemotherapies is imperative.

CSCs play a key role in cancer resistance to conventional chemotherapy 50. Here, we used a STAT3 inhibitor to block the activity of CSCs. We constructed NPs-Stattic-IL20RA, which carry the STAT3 inhibitor stattic and target the IL20RA-positive cells. NPs-Stattic-IL20RA combined with chemotherapies (DOX and CTX) and an αPD-L1 antibody effectively delayed the rate of tumor growth after treatment. This combination strategy thus enhanced the response to chemotherapy in IL20RA^hi^ breast cancer.

Although overexpression of IL20RA in breast tumor cells decreased the percentage of CD8^+^ T-cells in the tumor microenvironment, NPs-Stattic-IL20RA plus IgG did not increase the percentage of these cells compared with the IgG control. The underlying mechanism is complicated; one of the key reasons is that the amount of αIL20RA Ab used for NP preparation was very low (20 μg Ab per 1 mL NPs, 200 μL NPs/dose for treatment). In this study, the αIL20RA Ab was only used for targeting purposes to deliver stattic to the IL20RA^+^ tumor cells. As shown in **Figure S7B**, the αIL20RA antibody showed a minimal effect on the expression of downstream proteins, such as p-STAT3 (Tyr705), SOX2, and PD-L1, in 4T1-IL20RA cells at the relatively low concentration of 2.5 μg/mL and 5 μg/mL.

In conclusion, we found that IL20RA could regulate the stemness of breast cancer cells and promote a cancer-favorable immune microenvironment. Combination of chemotherapy with anti-PD-L1 antibody and NPs-Stattic-IL20RA provided a promising strategy for the treatment of IL20RA^hi^ breast cancer patients.

## Supplementary Material

Supplementary figures and tables.
