# Supplementary material for: IL20RA signaling enhances stemness and promotes the formation of an immunosuppressive microenvironment in breast cancer
Source: Theranostics. 2021 Jan 1;11(6):2564–80. doi: 10.7150/thno.45280 (PMC7806486; doi:10.7150/thno.45280)
Supplement: Supplementary file 1 — Supplementary figures and tables. [file thnov11p2564s1.pdf]

IL20RA signaling enhances stemness and promotes the formation of an immunosuppressive microenvironment in breast cancer

Supplementary Information

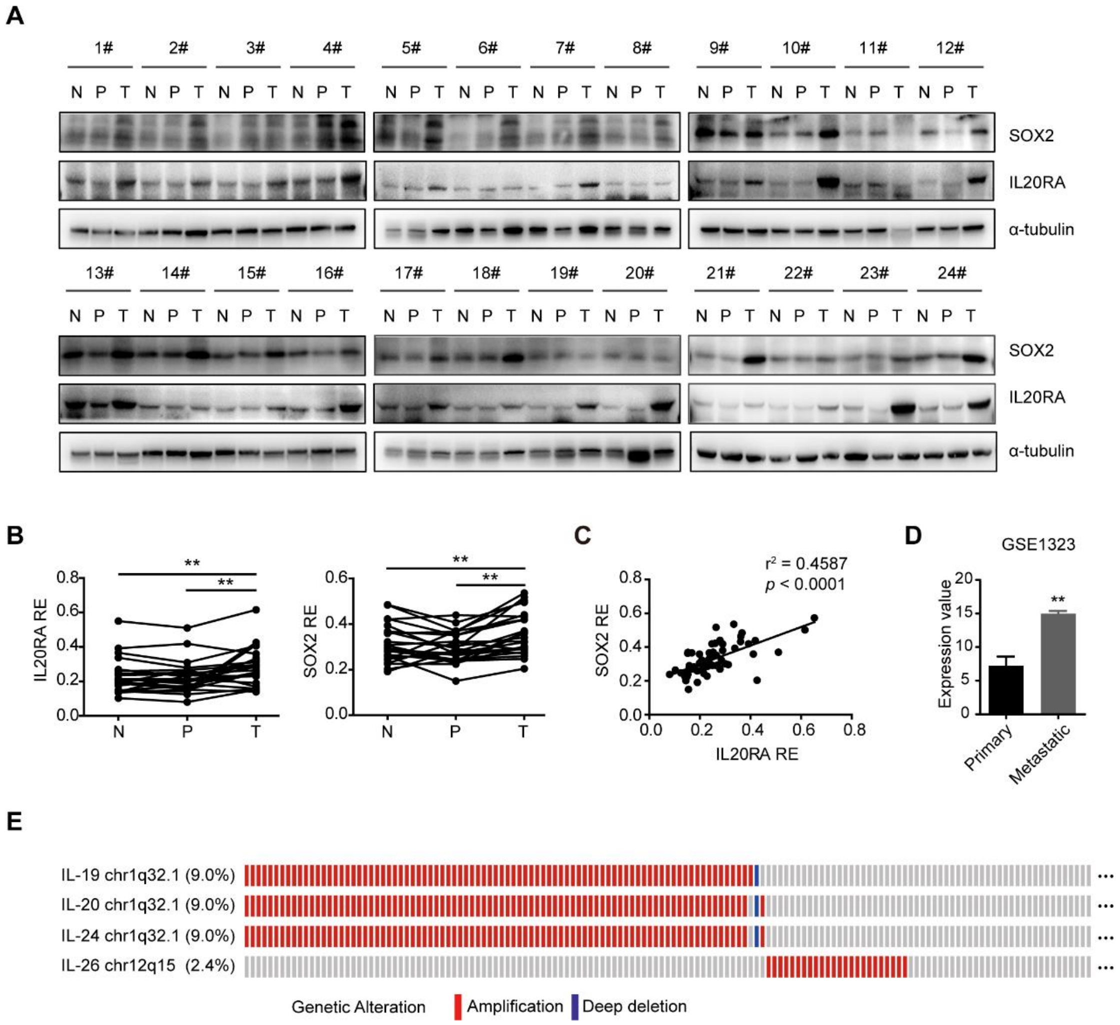

Figure S1. IL20RA is highly expressed in human colorectal cancers and correlated with the expression of SOX2. (A) Western blot results of IL20RA and SOX2 in normal (N), para-carcinoma (P) and tumor (T) tissues of 24 human colorectal cancer patients. (**B**) Quantifications of the expression of IL20RA (n = 23) and SOX2 (n = 23) for panel A. **(C)** Pearson's analysis of the correlation between SOX2 and IL20RA in these human colorectal normal, para-carcinoma, and tumor tissues (n = 69). **(D)** The expression of *IL20RA* mRNA in cell lines derived from primary tumor and corresponding metastasis of colon cancer from GEO dataset (GSE1323; n = 3 for each group). **(E)** Oncoprints of *IL-19*, *IL-20*, *IL-24,* and *IL-26* alterations in 996 invasive breast carcinomas (TCGA; PanCancer Atlas). **, $p < 0.01$.

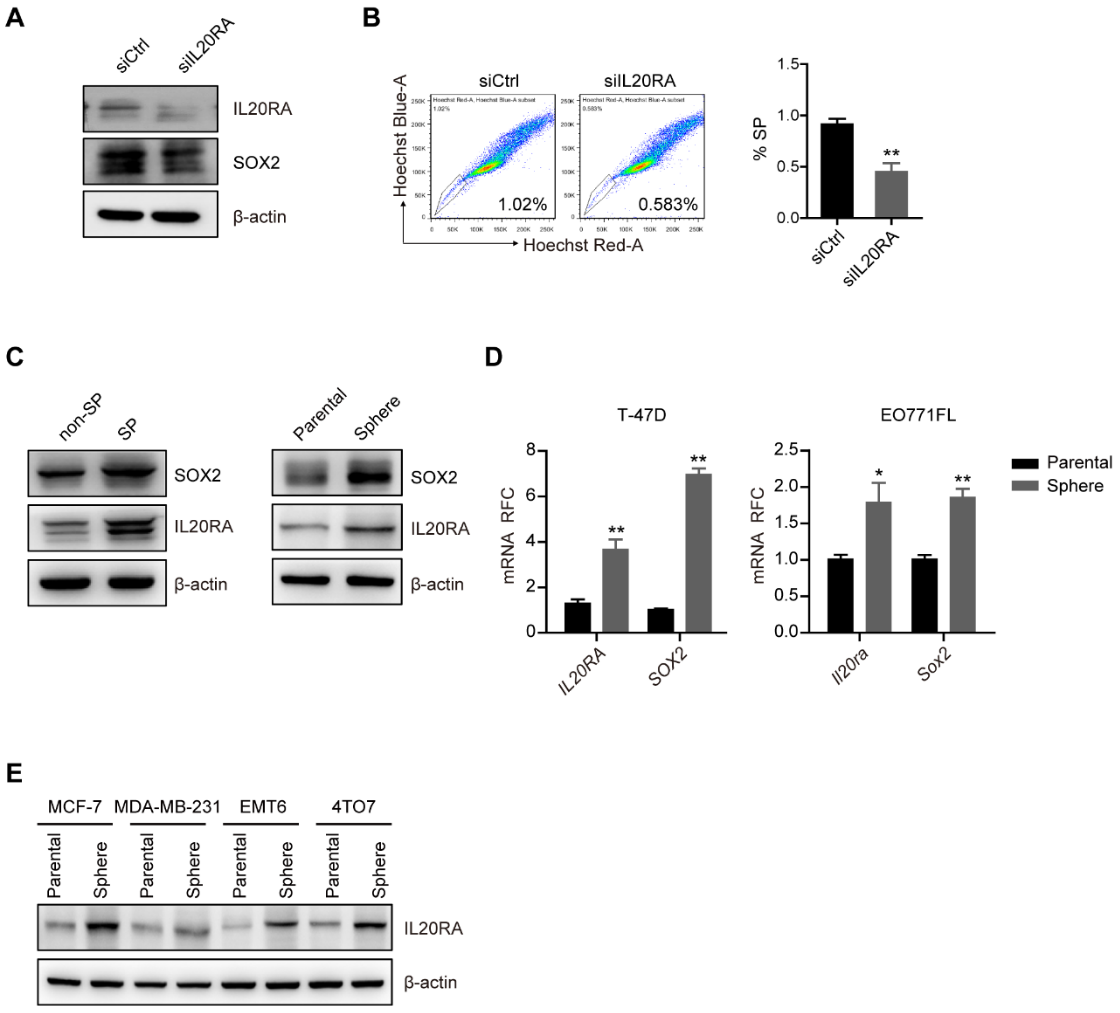

Figure S2. Elevated expression of IL20RA in CSCs-like cell populations of breast cancer. (A) Western blot results of IL20RA and SOX2 in MDA-MB-231 cells, which were transiently transfected with siIL20RA or siRNA control (siCtrl). **(B)** Flow cytometric analysis of SP in MDA-MB-231 cells, which were transiently transfected with siIL20RA or siCtrl. Left panel: representative flow cytometric analysis results. Right panel: statistical analysis of the SP proportions (n = 3 for each group). **(C)** Western blot results of SOX2 and IL20RA in non-SP and SP (left panel) and in parental cells and spheres (right panel) of EO771FL cell line. **(D)** Real-time PCR analyses of the relative fold changes of *IL20RA*/*Il20ra* and *SOX2*/*Sox2* mRNAs in spheres compared with their respective parental cells in T-47D and EO771FL cell lines, n = 3 for each group. **(E)** Western blot results of IL20RA in parental cells and spheres of different breast cancer cell lines. *, $p < 0.05$; **, $p < 0.01$.

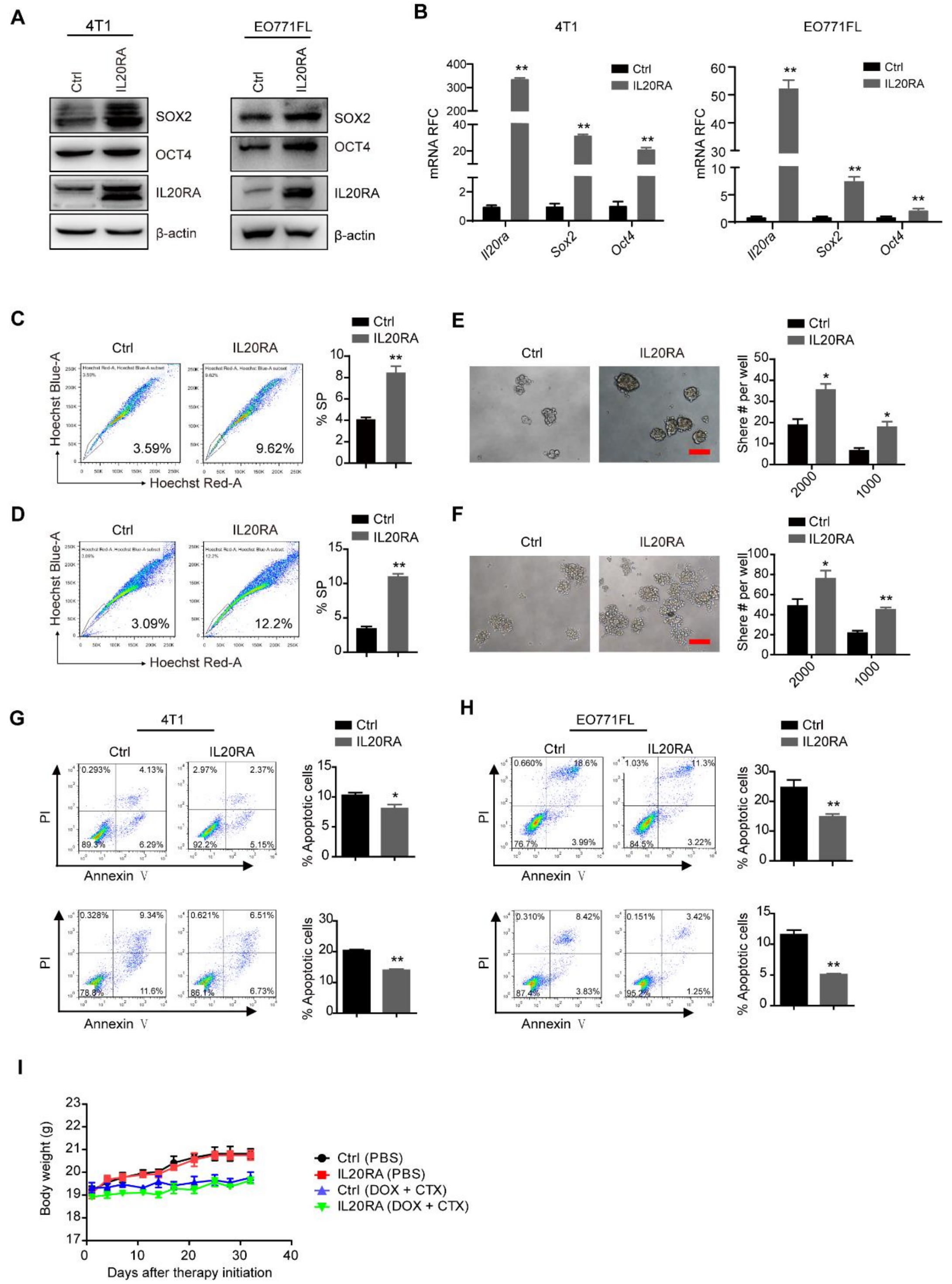

Figure S3. IL20RA promotes the stemness of breast cancer cells. (A) Western blot results of SOX2, OCT4, and IL20RA in 4T1 and EO771FL cells. **(B)** Real-time PCR analyses of *Il20ra*, *Sox2*, and *Oct4* mRNAs in 4T1 and EO771FL cells (n = 3 for each group). **(C-D)** Flow cytometric analysis of SP in 4T1 cells (C) and EO771FL cells (D). Left panel: representative flow cytometric analysis results. Right panel: statistical analysis of SP proportions (n = 3 for each group). **(E-F)** Sphere formation assay in 4T1 cells (E) and EO771FL cells (F). Left panel: representative sphere images. Scale bar, 100 μm. Right panel: statistical analysis of sphere

number (n = 3 for each group of 4T1 cells, n = 4 for each group of EO771FL cells). **(G-H)** Flow cytometric analysis of apoptotic cells in 4T1 and EO771FL cells, which were treated with DOX (upper panel) or CTX (lower panel). Left panel: representative flow cytometric analysis results. Right panel: statistical analysis of the percentage of apoptotic cells (n = 3 for each group). **(I)** Body weight curves of BALB/c mice bearing 4T1-allografts, which were treated with DOX plus CTX or PBS. Data are presented as mean ± SEM (n = 6 mice for each group). *, $p < 0.05$; **, $p < 0.01$.

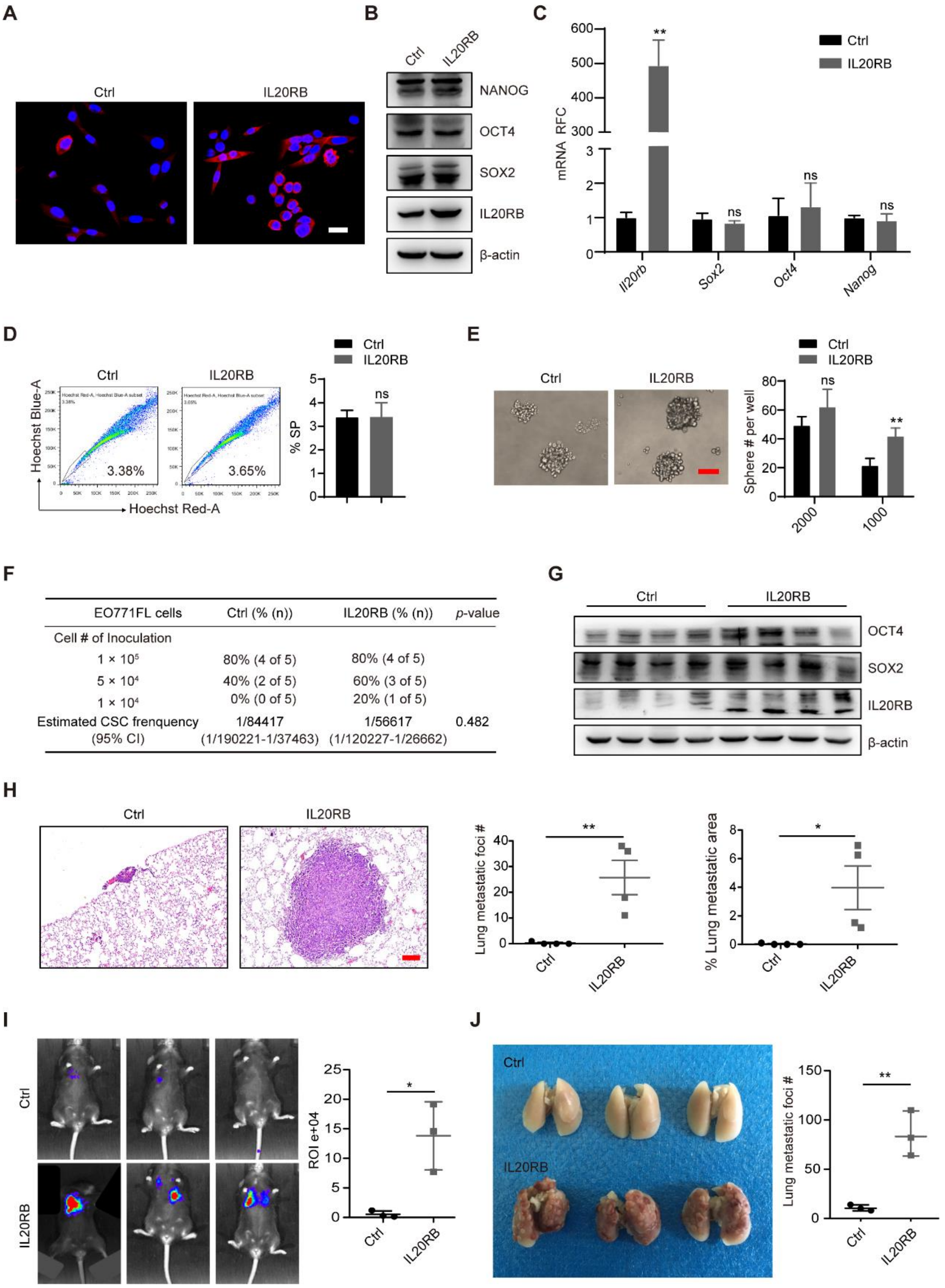

Figure S4. IL20RB has less impact on the stemness features, but promotes the lung metastasis of breast cancer cells. (A) Immunofluorescence staining of IL20RB in EO771FL cells. Scale bar, 20 μm. **(B)** Western blot results of NANOG, OCT4, SOX2, and IL20RB in EO771FL cells. **(C)** Real-time PCR analysis of *Il20rb*, *Sox2*, *Oct4*, and *Nanog* mRNAs in EO771FL cells (n = 3 for each group). **(D)** Flow cytometric analysis of SP in EO771FL cells.

Left panel: representative flow cytometric analysis results, right panel: statistical analysis of SP proportions (n = 3 for each group). **(E)** Sphere formation assay in EO771FL cells. Left panel: representative images of tumor spheres. Right panel: statistical analysis of sphere number (n = 4 for each group). Scale bar, 50 μm. **(F)** ELDA analysis of the tumor-initiating ability of EO771FL cells, which were injected into the 4th mammary fat pad of C57BL/6 mice. **(G)** Western blot results of OCT4, SOX2, and IL20RB in EO771FL allograft tissues. **(H)** Left panel: representative H&E staining images of lung metastasis in EO771FL-allograft mice. Right panel: statistical analyses of the metastatic foci number and the percentage of metastatic area of lungs. Data are presented as mean ± SEM (n = 4 mice for each group). Scale bar, 100 μm. **(I)** Bioluminescent images of lung metastasis of EO771FL cells 20 days after the tail-vein injection (left panel) and statistical analysis of normalized BLI signals (right panel; data are presented as mean ± SEM; n = 3 mice for each group). **(J)** Left panel: the lungs of mice at the end of experiments. Right panel: statistical analysis of lung metastatic foci number. Data are presented as mean ± SEM (n = 3 mice for each group). *, $p < 0.05$; **, $p < 0.01$; ns, not significant.

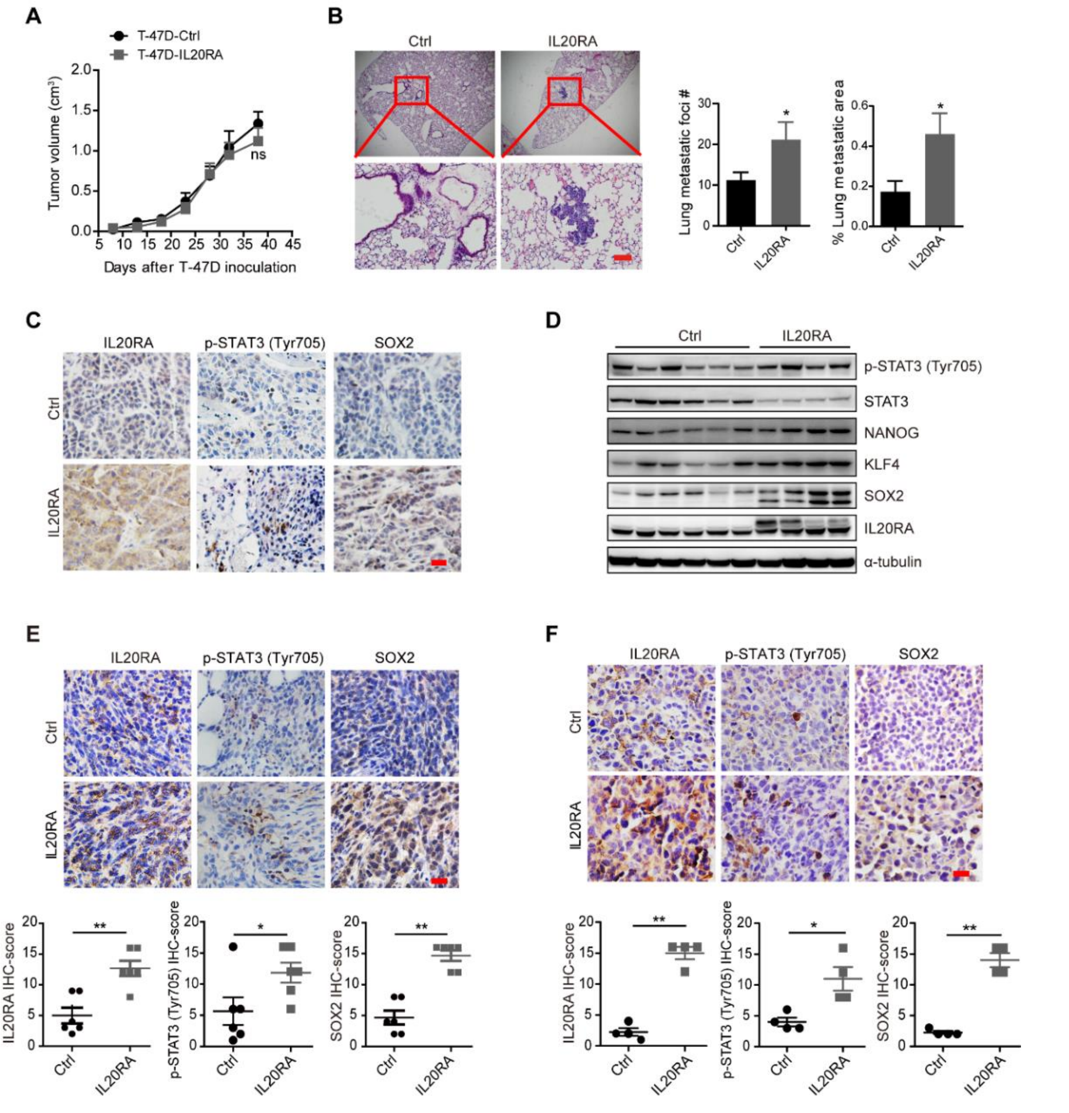

Figure S5. IL20RA activates STAT3 to promote the stemness of breast cancer cells. (A) Tumor growth curves of NOD/SCID mice bearing T-47D xenografts (n = 6 mice for each group). **(B)** Left panel: representative H&E staining images of lung metastasis. Scale bar, 100 µm. Right panel: statistical analyses for the metastatic foci number and the percentage of metastatic area of lungs (n = 6 mice for T-47D-Ctrl inoculation group, n = 4 mice for T-47D-IL20RA inoculation group). **(C)** Representative IHC staining images of IL20RA, p-STAT3 (Tyr705), and SOX2 in paraffin-embedded tissue sections of T-47D xenografts. Scale bar, 20 µm. **(D)** Western blot results of p-STAT3 (Tyr705), STAT3, NANOG, KLF4, SOX2, and IL20RA in T-47D xenograft tissues. **(E-F)** IHC staining assay of IL20RA, p-STAT3 (Tyr705), and SOX2 in paraffin-embedded tissue sections of 4T1 (E) and EO771FL (F) allografts. Upper panel: representative IHC staining images, scale bar, 20 µm. Lower panel: statistical analyses of the IHC-scores for IL20RA, p-STAT3 (Tyr705), and SOX2. Data are presented as mean ± SEM (n = 6 mice for each group of 4T1 allografts, n = 4 mice for each group of EO771FL allografts). *, $p < 0.05$; **, $p < 0.01$; ns, not significant.

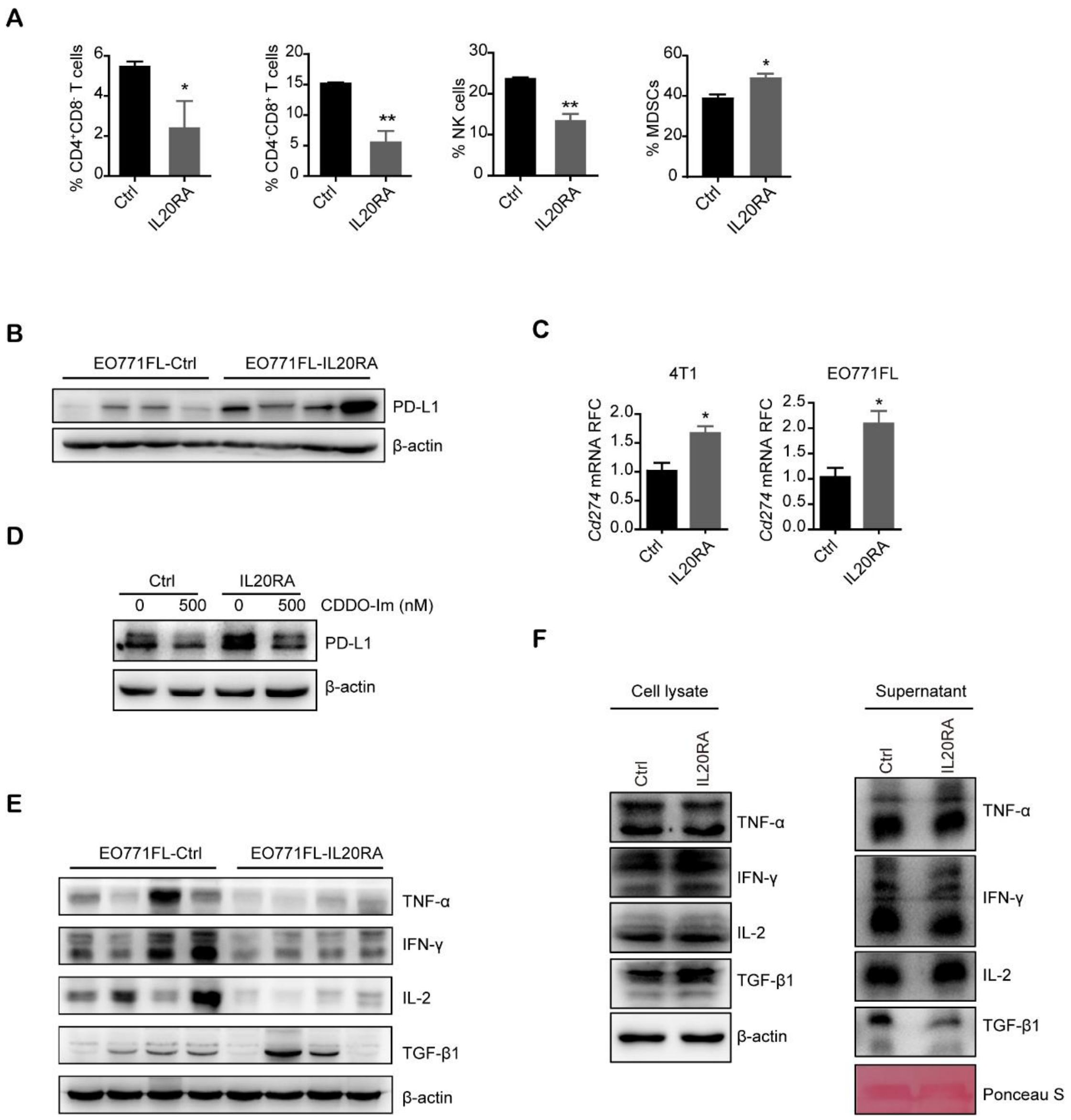

Figure S6. IL20RA promotes the formation of an immunosuppressive microenvironment in EO771FL allografts. (A) The proportions of TILs in the EO771FL allograft tissues were analyzed by flow cytometry. Cell populations were identified as $CD4^+$ T cells ($CD45^+CD4^+CD8^-$), $CD8^+$ T cells ($CD45^+CD4^-CD8^+$), NK cells ($CD45^+NK1.1^+$), MDSCs ($CD45^+CD11b^+Gr1^+$). Statistical analyses for the proportion of TILs in $CD45^+$ cells are shown (n = 3 for each group). **(B)** Western blot results of PD-L1 in EO771FL allograft tissues. **(C)** Real-time PCR analyses of *Cd274* mRNA in 4T1 and EO771FL cells (n = 3 for each group). **(D)** Western blot results of PD-L1 in T-47D cells, which were treated with or without CDDO-Im for 48 h. (**E**) Western blot results of TNF-α, IFN-γ, IL-2, and TGF-β1 in EO771FL-allograft tissues. **(F)** Western blot results of TNF-α, IFN-γ, IL-2, and TGF-β1 in cell lysate (left panel) and supernatant (right panel) of 4T1 cells. *, $p < 0.05$; **, $p < 0.01$.

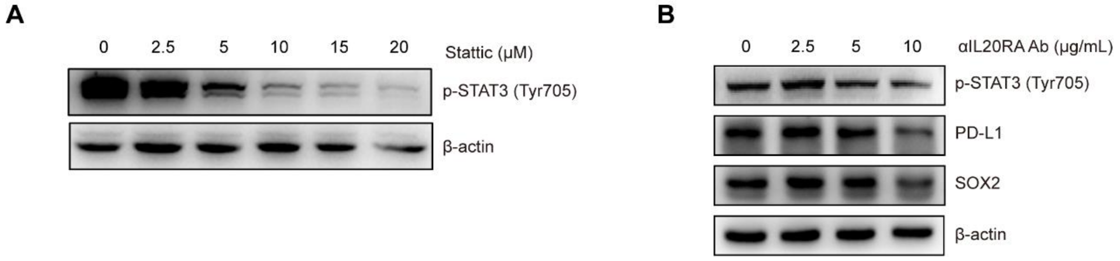

Figure S7. Stattic and αIL20RA Ab block the STAT3 signaling in a dose dependent manner. (A) Western blot results of p-STAT3 (Tyr705) in 4T1-IL20RA cell line, which was treated with different concentrations of stattic for 24 h. (**B)** Western blot results of p-STAT3 (Tyr705), PD-L1, and SOX2 in 4T1-IL20RA cell line, which was treated with different concentrations of αIL20RA Ab for 24 h.

A

Liposome	Size (nm)	Polydispersity index	Zeta potential (mV)
NPs-DOX	100.3 ± 1.935	0.251 ± 0.01	-8.47 ± 1.222
NPs-DOX-IL20RA	121.3 ± 1.769	0.178 ± 0.019	-5.61 ± 0.461
NPs-Stattic	117.9 ± 0.833	0.143 ± 0.051	-19.56 ± 4.902
NPs-Stattic-IL20RA	120.4 ± 2.910	0.231 ± 0.033	-11.3 ± 0.092

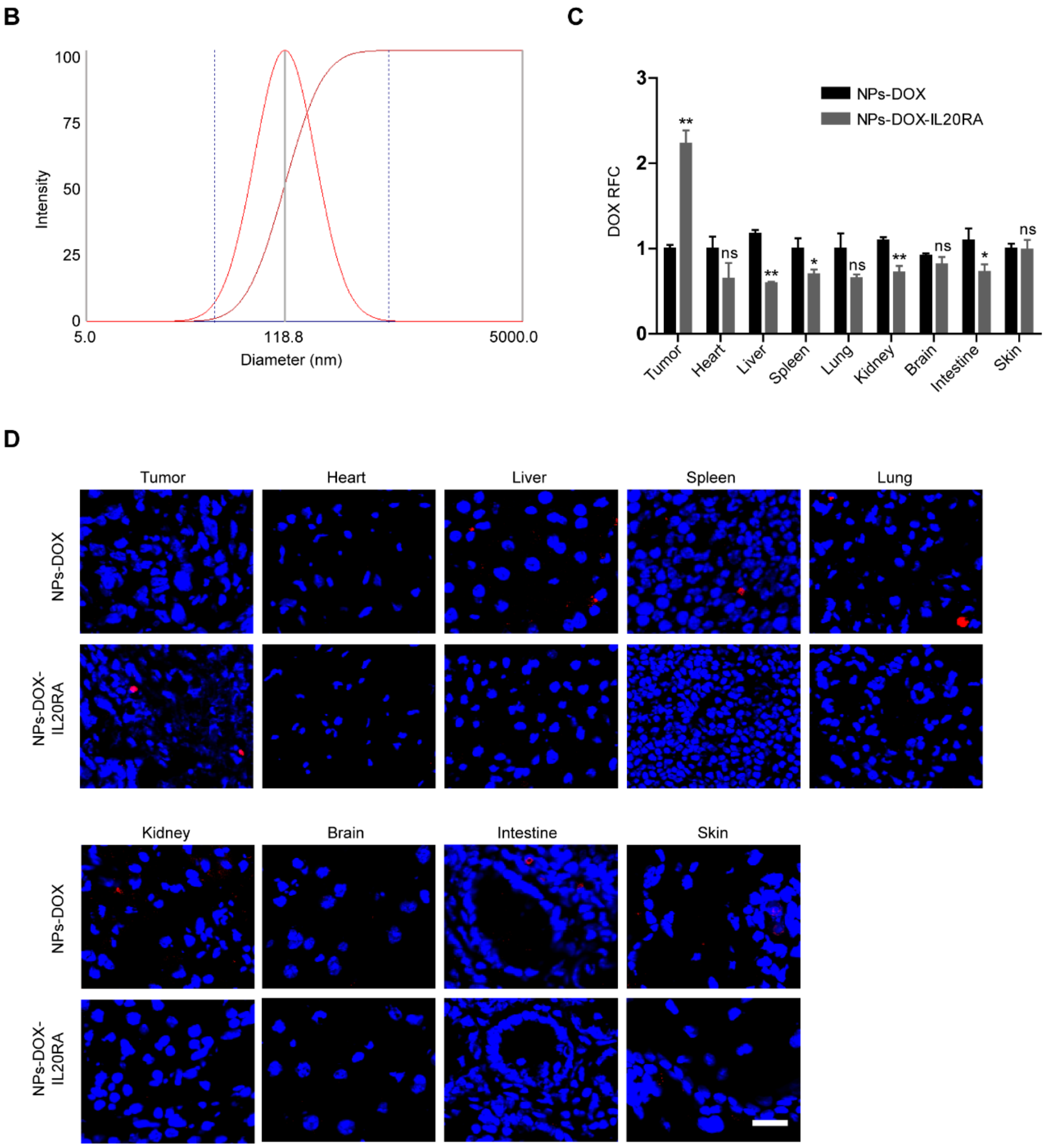

Figure S8. Characterization of IL20RA targeted liposomal nanoparticles. (A) The characterizations of nanoparticles. **(B)** Representative dynamic light scattering measurement of NPs-Stattic-IL20RA. **(C-D)** BALB/c mice bearing 4T1-IL20RA allografts were treated with NPs-DOX or NPs-DOX- IL20RA for 4 h. **(C)** The relative fold changes of DOX concentration in various tissues of BALB/c mice treated with NPs-DOX-IL20RA compared with the NPs-DOX treatment group (n = 4 for each group). **(D)** Representative confocal images of DOX in various tissues of BALB/c mice. Scale bar, 20 μm. *, $p < 0.05$; **, $p < 0.01$; ns, not significant.

Table S1. Clinical characteristics of breast cancer patients.

Number	Gender	Age	SBR Grade	Diagnosis
1	F	55	II	Invasive carcinoma
2	F	35	II	Invasive carcinoma
3	F	44	II	Invasive carcinoma
4	F	45	III	Invasive carcinoma
5	F	61	II	Invasive carcinoma
6	F	55	III	Invasive carcinoma
7	F	48	II	Invasive carcinoma
8	F	38	II -III	Invasive carcinoma
9	F	53	III	Invasive carcinoma
10	F	67	II	Invasive carcinoma

Abbreviations: F, female; SBR, Scarff-Bloom-Richardson.

Table S2. Clinical characteristics of colorectal cancer patients.

Number	Gender	Age	Site	Diagnosis
1	F	77	Rectum	Moderately-poorly differentiated ADC
2	M	52	Rectum	Moderately differentiated ADC
3	M	74	Rectum	Highly-moderately differentiated ADC
4	F	66	Sigmoid colon	Moderately differentiated ADC
5	M	53	Ascending colon	Mucinous ADC
6	F	68	Rectum	Moderately differentiated ADC
7	M	62	Rectum	Moderately-poorly differentiated ADC
8	M	74	Sigmoid colon	Moderately differentiated ADC
9	F	58	Sigmoid colon	Moderately differentiated ADC
10	M	39	Transverse colon	Mucinous ADC
11	F	65	Ileum	Mucinous ADC
12	M	67	Ascending colon	Moderately differentiated ADC
13	M	49	Ascending colon	Moderately differentiated ADC
14	M	75	Rectum	Moderately-poorly differentiated ADC
15	M	61	Rectum	Moderately differentiated ADC
16	M	66	Rectum	Moderately differentiated ADC
17	F	34	Rectum	Signet ring cell carcinoma
18	F	65	Rectum	Moderately differentiated ADC
19	M	62	Rectum	Poorly differentiated ADC
20	M	49	Rectum	Moderately-poorly differentiated ADC
21	M	60	Sigmoid colon	Moderately differentiated ADC
22	M	58	Rectum	Moderately differentiated ADC
23	F	75	Ascending colon	Moderately-poorly differentiated ADC
24	M	65	Rectum	Mucinous ADC

Abbreviation: M, male; F, female; ADC, adenocarcinoma.

Table S3. Clinical characteristics of healthy female donors and female patients with breast cancer in the ELISA assay of serum IL-19, IL-20, IL-24, and IL-26.

	**Normal (n = 18)**	**Non-metastatic BC (n = 27)**	**Metastatic BC (n = 27)**
**Age**			
26-35	0 (0%)	2 (7.41%)	0 (%)
36-45	6 (33.33%)	11(40.74%)	12 (44.44%)
46-55	12 (66.67%)	13 (48.15%)	15 (55.56%)
56-70	0 (0%)	1 (3.70%)	0
**Metastatic site**			
Bone w/wo other sites	NA	NA	13 (48.15%)
other sites (LN(s), liver, lung, etc.)	NA	NA	14 (51.85%)

Abbreviation: BC, breast cancer; w/wo, with/without; LN(s), lymph node(s); NA, not applicable.

Table S4. The siRNAs used for cell transfection.

siIL20RA#1	5'- GAUAUGAGUUUACUGGGAAdTdT-3'
	5'- UUCCCAGUAAACUCAUAUCdTdT-3'
siIL20RA#2	5'- GUGUUGAAUACUAAAUCAAdTdT-3'
	5'- UUGAUUUAGUAUUCAACACdTdT-3'
siIL20RA#3	5'- CAGUAUUAUGCCAAAGUUAdTdT-3'
	5'- UAACUUUGGCAUAAUACUGdTdT-3'
siCtrl	5'- UUCUCCGAACGUGUCACGUTT-3'
	5'- ACGUGACACGUUCGGAGAATT-3'

Table S5. The primers used for real-time PCR.

mus Sox2	Forward primer: 5'- ACAGCATGTCCTACTCGCAG -3'
	Backward primer: 5'- ATGCTGATCATGTCCCGGAG -3'
mus Oct4	Forward primer: 5'- TGGCTTCAGACTTCGCCTTC -3'
	Backward primer: 5'- TGGAAGCTTAGCCAGGTTCG -3'
mus Nanog	Forward primer: 5'- GAGTGTGGGTCTTCCTGGTC -3'
	Backward primer: 5'- GTCTTCAGAGGAAGGGCGAG -3'
mus Cd274	Forward primer: 5'- GAATTTCCGTGGATCCAGCC -3'
	Backward primer: 5'- ACTTCTCTTCCCACTCACGG -3'
mus Il20ra	Forward primer: 5'- TATTGCCCTGACAGCACCAG -3'
	Backward primer: 5'- GTGTGCTGTTGGTGACACAC -3'
mus Il20rb	Forward primer: 5’- TCACTGAACATGGGCTGCAT -3’
	Backward primer: 5’- TGCGCCTTCACACAGTACAT -3’
mus Gapdh	Forward primer: 5'- GGAGAGTGTTTCCTCGTCCC -3'
	Backward primer: 5'- ATGAAGGGGTCGTTGATGGC -3'
homo SOX2	Forward primer: 5’- GGATAAGTACACGCTGCCCG -3’
	Backward primer: 5’- ATGTGCGCGTAACTGTCCAT -3’
homo OCT4	Forward primer: 5’- GCTCGAGAAGGATGTGGTCC -3’
	Backward primer: 5’- CGTTGTGCATAGTCGCTGCT -3’
homo IL20RA	Forward primer: 5'- GACGGGCAGTTCCCTGTG -3'
	Backward primer: 5'- ACCCTCTGGTGGAGTCCATT -3'
homo GAPDH	Forward primer: 5’- CTCTGATTTGGTCGTATTGGG -3’
	Backward primer: 5’- TGGAAGATGGTGATGGGATT -3’
